# Internationally educated nurses and resilience: A systematic literature review

**DOI:** 10.1111/inr.12787

**Published:** 2022-07-22

**Authors:** Kari Dahl, Line Nortvedt, Judith Schrøder, Ann Kristin Bjørnnes

**Affiliations:** ^1^ Oslo Met, Oslo Metropolitan University Oslo Norway

**Keywords:** Culture, Immigration International Issues, International Collaboration/Cooperation International Issues, Literature Reviews Research, Nursing Education Nursing

## Abstract

**Aim:**

To synthesize knowledge extracted from the literature about protecting factors and challenges to resilience, among migrant nurses, and specifically how knowledge synthesized through the process of the literature review is relevant to nursing and health policy.

**Background:**

How nurses, in general, face challenges is well documented and is often linked to the concept of resilience; however, there seems to be a lack of systematic knowledge synthesis focusing on the resilience of internationally educated nurses following migration.

**Method:**

The review was guided by the PRISMA guidelines, and a systematic search of peer‐reviewed qualitative and mixed‐method articles reporting empirical research was performed in the MEDLINE, CINAHL, PsycINFO and Academic Search Ultimate databases. Methodological rigour was assessed by the Joanna Briggs’ checklist, and a structured theme‐based ecological framework, inspired by Ungar's model of resilience, was chosen.

**Results:**

Following critical appraisal, 37 studies were included that identified both challenges and individual, contextual and structural protective factors in host countries and are linked to resilience.

**Discussion:**

Resilience of internationally educated nurses depends on a combination of individual and contextual protective factors, with the major emphasis being placed on individual protective factors. It is crucial to consider resilience in ensuring that internationally educated nurses’ experience is appreciated, as this is necessary if nurses are to deliver the best possible health service while integrating into their host country.

**Implication for nursing and health policy:**

Authorities, managers in clinical practice and education, trade unions and nurses in general should be aware of the coping strategies, the strengths and supportive factors that can promote resilience and be aware of the challenges that undermine resilience and negatively impact internationally educated nurses’ practice and social interactions.

## INTRODUCTION

Globally, internationally educated nurses (IENs) make an important contribution to the workforce, bringing important skills to healthcare systems around the world. IENs are individuals who have completed a nursing education programme that is not located within the employer country (National Council of State Boards of Nursing, 2016). Research indicates that the motivation behind migration primarily involves financial circumstances, but it also derives from social, professional and personal factors, with the overall goal of better career opportunities and improved quality of life (Dahl et al., 2021).

Structural factors, such as political instability, violence and crime in the country, of origin, are other important drivers of migration (Pung & Goh, 2017). Nurses who originate from India and the Philippines form the largest source of foreign labour in the global north (Thompson & Walton‐Roberts, 2019). Approximately 16% of all nurses working in various Organization for Economic Co‐operation and Development countries are foreign‐born (Socha‐Dietrich & Dumont, 2021).

Evidence from research studies has shown that IENs experience challenges in their host countries following migration, including a lack of professional development opportunities (Chun Tie et al., 2019) and the perception of being devaluated when their nursing competence and skills are under‐rated (Dahl et al., 2017; Nortvedt et al., 2020). IENs miss their friends and family at home (Alexis & Shillingford, 2012) and have difficulties orienting themselves to the new country's governmental requirements concerning their authorization as nurses (Eriksson et al., 2018). Communication barriers and experiences of discrimination and marginalization are also highlighted as individual challenges (Magnusdottir, 2005). All these challenges can create emotional stress and frustration and lead to a sense of isolation and loneliness; however, they can also stimulate resilience, and the majority of IENs choose to stay.

How nurses, in general, face challenges in their education and work is well documented and is often linked to resilience, a concept for which there is no single, definition (Niitsu et al., 2017). There is a lack of systematic knowledge synthesis focusing on the resilience of IENs (Aburn et al., 2016). Bolton et al. (2017) describe resilience as the ability to bounce back or recover from adverse conditions, and Stacey and Cook (2019) explore the concept of resilience in nursing further and claim that resilience focuses on the individual nurse's self‐efficacy, subjective motivation to cope and capacity to respond confidently to difficult situations. Niitsu et al. (2017) conducted a concept analysis of resilience as linked to probably traumatic incidents, and they concluded that ego‐resilience, emotion regulation, social backing and heredity were defining attributes and important to an understanding of resilience among nurses.

In the current study, we aim to expand on these ideas and to explore resilience from the interactive person–environment process perspective that is inspired by Michael Ungar's ecological understanding of resilience, in which the interaction between living organisms and their environment is central (Ungar, 2014). According to Ungar's definition

‘Resilience is the ability of individuals (on their own or collectively) to navigate to the culturally relevant resources they need to do well when confronting adversity, as well as their capacity to negotiate for these resources to be provided in ways that are meaningful.’ (Ungar, 2014, p. 41)

The resources described by Ungar can be individual, relational or collective, and these protective contextual factors are dependent on family influences that are both environmentally and genetically mediated (Ungar, 2014).

### Aim of the study

The aim of the study is to synthesize knowledge extracted from the literature about protective factors and challenges to resilience, how resilience manifests among and benefits migrant nurses, and specifically how knowledge synthesized through the process of the literature review is relevant to nursing and health policy. The objectives were (i) to describe resilience as connected to the personal and interpersonal characteristics of IENs and their new work environment and (ii) to explore what protecting factors (resilience) IENs apply when facing challenges in their host countries.

## METHODS

This study was guided by the PRISMA guidelines (preferred reporting items for systematic reviews and meta‐analyses) to ensure methodological quality (Page et al., 2021) (Supplementary Table S1). A review protocol was created, and the review was registered and confirmed by Prospero, which is an international prospective register of systematic reviews.

### Selection criteria

A systematic search was conducted in June 2020 to assess the range and nature of knowledge and concepts relating to resilience and IENs. The MEDLINE, CINAHL, PsycINFO and Academic Search Ultimate databases were systematically searched in collaboration with a research librarian. Inclusion criteria were purposefully kept broad to gain a comprehensive understanding of resilience. The study comprised primary qualitative and mixed‐method articles, published in English or in a Scandinavian language in a peer‐reviewed journal between 2005 and 2020. The publication period is based on the changes in requirements for nursing education established by the Bologna process, which is a series of agreements between European countries to ensure comparability in the standards and quality of higher‐education qualifications, and the increasing migration and demand of nurses specially in the Global North from the early 2000s (European Education Area, 2022; Socha‐Dietrich & Dumont, 2021).

Keywords and MeSH terms were systematized by applying a modified SPIDER, in accordance with the research question and the study eligibility criteria, which included sample, phenomenon of interest, design, evaluation and research type which appears in Table 1 (Cooke et al., 2012). The selection of search terms relating to the phenomenon of interest was influenced by the Ungar definition of resilience and by advice from our research librarian concerning related search terms as indexed in the various databases.

### Data selection and data extraction process

**FIGURE 1:**
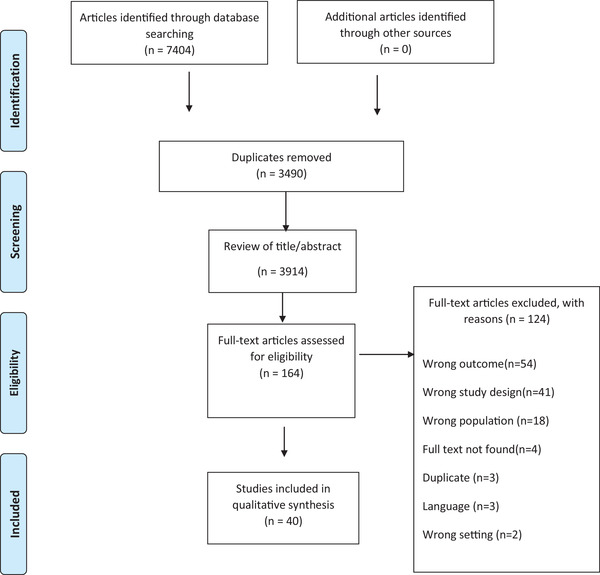
Prisma flow diagram

All four researchers were involved in the data selection and data extraction process, using the Covidence literature‐screening software, which is used for managing and streamlining the review process and allows for blinded double‐screening in all review phases. The first screening of potentially eligible studies was limited to titles and abstracts. The citations were imported into Covidence, 3,490 duplicates were removed, and a total of 3,914 titles and abstracts were double screened against eligibility criteria. A total of 164 articles were deemed to meet the inclusion criteria, full texts were retrieved and the articles were double screened again. In all phases, conflicts were resolved by discussion between the reviewers. Finally, 37 articles were included in the review (Figure 1). The methodological quality of each included study was independently assessed by two reviewers using the Critical Appraisal Skills Program JABRI (Joanna Briggs Institute, 2020). Any uncertainty regarding the methodological quality of a study was resolved through discussion among all the review authors.

Data extraction and quality assessment templates were created in Covidence, and information about year, country, aim, design, recruitment of participants, population characteristics, including sample size, sex, age, IENs’ country of origin, main findings (e.g. themes) and information about specific themes relating to resilience were collected. All information was independently extracted by two review authors and, thereafter, compared and crosschecked by the last author.

### Synthesis of findings

A structured theme‐based framework inspired by Ungar's model of resilience was chosen against which to analyse the qualitative findings of the articles included. The results sections of the included studies were uploaded to the computer software NVivo12 (NVivo, 2018; NVivo, Version12). In line with Elo and Kyngäs (2008), we used a three‐phase process that included preparation, organization and reporting. First, we identified the analytic units in the selected articles. Then, we coded and identified categories. Finally, the results were reported with links to the findings of prior knowledge. Following content analysis using a deductive approach (Elo & Kyngäs, 2008), the texts were coded into specific main themes in accordance with the factors referred to in Ungar's (2014) definition of resilience: individual resilience, contextual resilience, cultural, pluralistic and society‐supported resilience. Each of the four authors independently coded the extracted data and developed descriptive themes, primarily deductively. Consensus on data extraction included general information and the characteristics of the included studies, including consensus on quality assessment, and consensus meetings were held regularly to ensure eligibility of the articles. The studies were relatively homogeneous, and the concepts from the theoretical framework proved to be suitable for supporting analysis across the studies (Table 1).

Study results connected to the different concepts of resilience were divided into three main themes and five subthemes (ST): (1) Individual protective factors; ST: individual coping strategies, and hope, dreams, and strength; (2) contextual protective factors; ST: support from the family, and support in the workplace; and (3) structural protective factors; ST: support from society. Table 2 outlines the analytic process and presents representative quotes that emerged from the included studies.

**TABLE 1:** SPIDER table of the inclusion and exclusion criteria

**S**ample	Foreign‐educated nurses, foreign professional personnel, nurse
Phenomenon of interest	Migrate, emigrate, transnational, foreign, resilience, hardiness, adaption, coping, strength, self‐reliant, adjust, hardiness, positive mind, hope, security, faith, love, belong, empower, challenge, proud, courage and conscience
Design	Qualitative design
Evaluation	Not applicable
Research type	Qualitative methods and mixed methods

**TABLE 2:** Examples from the synthesis process

**From the article**	**Codes**	**Subtheme**	**Theme**
Learning different communication styles, support each other, breaking down language barriers and becoming more fluent, feeling and doing better (Jose, 2009). Coping strategies despite many barriers and sense of persistence: ironical sense of humour (Sochan & Singh, 2007).	Strength Hardiness Hope Dreams Positive mind Courage Self‐reliant Proud Faith	Individual coping strategies Hope, dreams and strength	Individual protective factors (resilience)
The core of Jordanian nurses’ social life is their family. Children were an opportunity for migrant nurses’ families to socialize and integrate with their local communities (Al‐Hamdan et al., 2015). The participants described a strong connection to their nuclear and extended families with a strong sense of duty to, and respect for, their families. They sought and received social support primarily from their family members and parents commonly live with their adult children and their families, sharing in childcare and household responsibilities (Connor, 2016).	Security Belong Mentoring Recognition Support Adapt	Support from the family Support at the workplace	Contextual protective factors (resilience)
Nurses and midwives coming from the EU found it quite easy to obtain a Swedish license (Eriksson et al., 2018). Participants identified a need for a supportive environment. Their recommendation that nursing education change to become more inclusive and supportive of minority students is a logical extension of the value they gave to diversity.	Role as a nurse Competence Affiliation Transition	Support from society	Structural protective factors (resilience)

## RESULTS

Supplementary Table S2 outlines the characteristics of the 37 included studies and provides an overview of the descriptions and concepts relating to resilience as found in each study. The studies were relatively homogeneous over the time period of publication, and no pattern related to time was observed. Most of the studies were conducted in the USA (*n* = 10), the UK (*n* = 6), Canada (*n* = 5) and Australia (*n* = 5), in addition to studies from New Zealand (*n* = 4), Sweden (*n* = 2), Iceland (*n* = 1), Singapore (*n* = 1), Chile (*n* = 1), Norway (*n* = 1) and Germany (*n* = 1).

A total of 1,501 informants were included across the studies (range 1–342), with the majority being female. The informants were IENs originating from all five continents, with approximately 50% originating from India and the Philippines. One study included native nurses, and two studies included nurse managers. Most of the studies utilized either a phenomenological, hermeneutic, exploratory or descriptive qualitative research design.

It became evident that none of the studies had a clear description of resilience; however, rich descriptions of related themes emerged from across the studies included. Unsurprisingly, dense descriptions of the IENs’ challenges were found. Most frequently described were challenges relating to language, which affected both the IENs’ clinical practice and their social interactions. Limited language skills, unfamiliarity with medical terminology, slang, varying accents, pronunciations, spelling, colloquialisms, speed of speech and telecommunication all generated uncertainty in interactions with colleagues and patients and made it easy to blame the IENs if errors occurred (Lin, 2014; Xu et al., 2008).

This was a phenomenon that occurred not only with IENs from countries speaking other languages but also for IENs who migrated from countries that spoke the same language (Chun Tie et al., 2019). Other challenges were associated with social adjustments, which included gender roles, cultural practices such as arranged marriages, non‐Christian religious beliefs, non‐verbal behaviours such as when to smile and how to make adequate eye contact and experiences of racism (Iheduru‐Anderson & Wahi, 2018). These cultural challenges related to both the general society and the healthcare context (Bland & Woolbridge, 2011; Salma et al., 2012; Stubbs, 2017).

### Individual protective factors

Individual resilience is defined by individually focused factors that contribute to advancement under stress, such as self‐efficacy, self‐esteem, positive peer relation and other protective factors that are centred on individual behaviour (Ungar, 2014). According to Ungar coping, adaptation, belief systems, self‐worth and character strength are all important factors that dictate how people will respond to stress or adversity (Theron et al., 2022).

#### Individual coping strategies

Different individual coping strategies were described in detail in about 50% of the studies, and these were linked to how the IENs cope with the challenges caused by stress relating to, for example, language difficulties, discrimination and complicated and expensive nurse licensure processes (Connor, 2016; Foong et al., 2005;Salami et al., 2018; Wheeler et al., 2014). The coping strategies, indicating resilience, are often intended to deal with being accepted in a new country and having positive relationships with others. The coping strategies often involve learning the community's system and different communication styles and attitudes, having faith, using an ironic sense of humour and making jokes and also asking for support from family, friends, church, colleagues and patients (Connor, 2016). These strategies are described as important in enabling IENs to be strong and to develop a sense of persistence despite the challenges, and they indicate their courage and awareness of possibilities. Individual characteristics such as optimism, faith, positive attitudes, not giving up and standing up for oneself are described in several of the articles and are evident in this quote:

*‘*I know that God prepared me before he sent me here …. . like I said, it's like survival of the fittest’. (Connor, 2016, p. 198)

In line with Ungar's (2014) thoughts about belief systems as being part of resilience, many of the articles highlight the importance of belief, of being respected for one's religious faith with the possibility of praying and being prayed for, of freedom of religion, and of meeting with people you can relate to. Connor (2016) also describes an ‘avoidance coping strategy’, as this quotation from an individual interview expresses.

‘You don't want to answer them in a crazy way, so you go someplace [sighs] and let it out because it's hard. I just go to staff lounge, drink some water and come back…’ (Connor, 2016, p. 199).

Adaptation to a new culture and context is described in several of the articles, and the importance of maintaining one's own cultural identity and sense of self‐worth is highlighted as an important protective factor when adapting to a new culture. Other examples of resilience are the willingness to adapt and learn to enable a smooth transition through the nursing career (Philip et al., 2019) and to fit in culturally, which has been described as ‘learning Australianisms’ as being invited to a friend's house and being asked to ‘bring a plate’ (Vafeas & Hendricks, 2018, p. 92).

Another central protective individual factor involves the process of adapting to the physical distance from family and culture (Jenkins & Huntington, 2016). The adaptation process is often described as seeking different experiences and further education, and this attitude is another important protective individual factor (Kishi et al., 2014).

#### Hope, dreams and strength

Other protective individual factors that can be linked to resilience are hope and dreams for oneself and one's family, including the possibility of a better financial and social life, a future, and opportunities to travel home. Lenz (2021) underlines that the expression of social hope, happiness with a focus on the future, plays a significant role in resilience among youth. Before migration, media as television in their home counties invariably led IENs to believe in their dreams of a better life: a place where they could be happy without struggles. Promises of earning a lot of money and being able to help others were also highlighted (Adhikari, 2013; Jose, 2011). One article describes a Filipino nurse who had dreams of starting a new business, after being a nurse and making money in a foreign country.

‘I want to start a business back home as soon as I have enough money, and as soon as I have gained enough experience here….’ (Alonso‐Garbayo & Maben, 2009, p. 4)

Having a strong belief with optimistic feelings about what the future will bring once they have completed their registration as a nurse seems to be important (Bland & Woolbridge, 2011, p. 23). Strength as an individual protective factor is described in many articles, which emphasizes that character strength is built on inherent personal strength and involves positive optimistic feelings and self‐discipline. One participant said:

‘I got to be strong; I got to be tough; I got to overcome all the problems. If you can survive in your working place, you will also be happy in being here.’ (Xu et al., 2008, p. E40)

Individual strength is also described as being willing to learn, to create networks and to find support, to pray and rely on that inner confidence, to gain social recognition and respect and to ignore discrimination. Several of the articles highlight courage and pride as individual protective factors and emphasize that the IENs are proud of their knowledge, experience, capabilities, work capacity and the feeling of being needed at work. As one nurse said:

‘I think I'm academic and I think I have so many different experiences under my belt. I'm competent. I feel I'm able to manage and do a good job…’ (Eriksson et al., 2018, p. 6)

### Contextual protective factors

This theme considers the contextual protective factors linked to resilience, where family influences, both environmental and genetically mediated, are essential. According to Ungar (2014), these protective factors have an effect on peer groups and community cohesion.

#### Support from the family

Many IENs struggle with loneliness, lack of social support networks and feelings of isolation due to being separated from their family. The decision to move to another country with the aim of working there is often based on family need, and the IENs feel obligated to send their family monetary support (Ronquillo, 2012; Sochan & Singh, 2007; Walters, 2008). Family relations are associated with a strong sense of duty, but the family is also a place to go to when you need help, as this informant describes:

‘For the first time in my life, I felt that I was so important in my family, everybody, all my relatives were trying to help me in their own way.’ (Adhikari, 2013, p. 174)

The family, particularly the nuclear family, is described as being at the core of the nurse's social life, and when the participants were asked who they would speak to if a serious situation occurred (Al‐Hamdan et al., 2015).

Findings indicate that having a family in the host country changes the IEN's position and opportunities and makes it easier to become integrated and to socialize within the community, especially when there are children.

#### Support at the workplace

Support and constructive feedback from co‐workers and managers are urgent for adaptation and survival in the workplace (Wolcott et al., 2013). Furthermore, access to support, both from natives and from nurses with the same origins, seems to be an important protective factor, which has been shown to protect against mental disorder, to enable the IEN to thrive in the workplace and be able to handle the challenges. Seeking support from other nurses from the same origins is highlighted in some articles and is seen to make the nurses stronger. The following quotation underlines the importance of fellowship:

*‘*If she [a Korean nurse] was not there to listen to me, then I might have gone to a psychiatric hospital!’ (Yi & Jezewski, 2000, p. 724).

Being valued for one's own cultural competence also seems vital (Jose, 2009; Salma et al., 2012). Support through language and mentoring programmes and further education are all important to success in the workplace. The IEN acquires important empowerment through receiving mentorship and high‐quality education (Iheduru‐Anderson & Wahi, 2018; Rodriguez et al., 2014). Working conditions that emphasize respect for diversity and difference and being included in a community of practice are both highlighted (Allen, 2018; Ramji et al., 2018), although nurses have to work hard for this.

‘You actually have to work so hard to make them know that you exist on the unit.’ (Jose, 2011, p. 64), and *‘*I was lucky, because I had a good manager’ (Kanter, in Eriksson & Engstrom, 2018, p. 869).

These quotations underline that both individual strength and a supportive environment are important when facing challenges.

### Structural protective factors

According to Ungar (2010), cultural, pluralistic and society support is another important protective factor linked to resilience, which can be exemplified as a simplified authorization process, the experience of competence development, easily accessible information, good working conditions and a facilitated transition. Ungar highlights that the resilience of the family and its members depends just as much on the resilience of the society and the individuals therein.

#### Support from society

The credentialing process is often described as exhausting, inefficient, time‐consuming and expensive, and IENs frequently lack adequate knowledge about the often very complex credentialing process. In some countries, IENs need to pass a nursing licensure exam, several language tests, and hold a position in a hospital before they are able to apply for approval as a nurse (Liou & Cheng, 2011; Salami et al., 2018), and the finding does not indicate that the process has become easier over the selected time period. Our analysis shows that the credentialing process should be simplified, information should be more accessible, and a supportive environment in the society should be highlighted. In one study, however, nurses and midwives described obtaining a Swedish licence as easy and the process as quite transparent and straightforward (Eriksson et al., 2018).

Access to knowledge through their workplace intranet and websites and opportunities for advancement such as in‐service training and attending university courses were underlined as important protective and supporting factors (Eriksson & Engstrom, 2018; Winkelmann‐Gleed & Seeley, 2005).

Furthermore, better working conditions are highly valued (Bland & Woolbridge, 2011). One study highlights the participants’ positive attitudes towards moving from a monoculture society, with a hierarchical approach to authority and working style, to a society with a flattened approach to authority (Healee & Inada, 2016).

Many articles describe the working conditions of IENs as nurses in the new country by comparing their current clinical experiences with those in their previous nursing positions. The IENs emphasize the reduced workload, their role function within a more flattened approach to authority, greater clinical autonomy, better career prospects and possibilities of further education. As one Nigerian IEN said:

‘I love the profession. I think America is a good place for me to do it, to do a better job and to expand in my education.’ (Jose, 2009, p. 67)

The significantly lower workload with fewer patients and different role functions as spending more time on the computer compared with their experiences in their home country were confirmed by other IENs (Eriksson & Engstrom, 2018; Eriksson et al., 2018). Furthermore, being autonomous as a nurse, working in an interdisciplinary group and not being dependent on doctors are all expressed as positive factors for professional identity and holistic care.

‘….Back home, doctors run hospitals, I mean they are the owners of the hospitals and many directors and managements are doctors.’ (Choi et al., 2019, p. 8)

One study describes how joining transitional programmes and using prearranged accommodation with information about associated costs, food and travel costs are all important supportive factors for a successful transition in the migration process (Chun Tie et al., 2019; Stubbs, 2017). One article highlights that recruiting agencies can facilitate IENs’ transition by initiating early planning for the recruitment, by providing detailed information about the new culture and nursing practice and focus the necessity of regular evaluation and adapting recruitment strategies (Lin, 2014).

### Quality appraisal

Methodological rigour was assessed by applying the checklist for qualitative research methodologies developed by the Joanna Briggs’ Institute (2020). The methodological quality was diverse across the included records with variations in relation to transparency in most studies (Supplementary Table S3). Fifty per cent to 70% of the studies had insufficient details to permit judgements relating to whether the findings were influenced by the researchers’ cultural or theoretical backgrounds, and if the data were adequately aligned with the participant narratives. However, the research methodology, the research question or objectives, the data collection process and the data analysis were clearly described in approximately 70% of the studies. The ethical perspectives were clearly described in most studies, and the conclusions were clearly drawn from the findings in 83% of the studies, indicating their trustworthiness. Despite the broad search strategy, few studies explicitly applied the term resilience. However, the results presented rich data about related themes.

## DISCUSSION

The results of the studies reported in this literature review offer insight into how IENs have different personal characteristics within the individual, contextual and structural protective factors that enable them to respond to the various challenges they face in host countries. The results highlight that a combination of individual and contextual protective factors is used by the IENs, with the main emphasis being on the individual protective factors, which is in line with Ungar's view that resilience is often individualized (Ungar, 2014). However, it is problematic to transfer resilience as a concept to how the IENs meet their different challenges. In accordance with Abdolrahimi et al. (2017), a concept contains three components: antecedents, defining attributes and consequences. A concept is also constantly changing, shaped by cultural, contextual and societal factors. By applying this understanding to the concept of resilience, the antecedents, attributes and consequences might be interpreted as the individual, contextual and structural protective factors that will be influenced by societal and cultural factors. Whether, for instance, hope, dreams and strength (resilience) are personal attributes of IENs or whether they are the antecedents or consequences of challenges are difficult to determine. However, there is no doubt that all protective factors (resilience) are influenced by cultural, contextual and societal factors.

Despite individual perspectives and protective factors (i.e. resilience as a personal trait) being highlighted in the included studies, contextual and certain structural protective factors are also described as substantial in enabling the IEN to be resilient. In particular, support from the family and workplace is a significant protective factor when facing challenges in a new country and is almost as important as the individual factors enabling resilience. Family support, the expectation of sending money home to the family, and the remittance policy are well known in the literature that describes the migration of nurses (Singh, 2016). This can be linked to a cultural understanding of resilience, as helping the family will give the nurse strength. All the protective factors can be related to an ecological understanding of resilience, where the challenges and protective factors described by IENs in the included studies can all be related to different levels of the ecological system: individual, contextual and structural or cultural supportive factors all contribute to the nurse's resilience (Ungar, 2011, 2014).

However, support from society was less prominent in the studies included. Whether society takes any responsibility for assessing the various challenges of migrated nurses and for facilitating the exercise of their competence is less than clear. The relationship between the individual's responsibility and society's responsibility is discussed in articles that see resilience as critical. In these, the nurses are encouraged to embrace a larger political and organizational perspective on their resilience (Traynor, 2017).

### Motivation to remain in host country

The motivation behind migration described in these studies is consistent with other studies, which highlight that the motivation to migrate is often prompted by financial, professional, political, social or personal circumstances (Pung & Goh, 2017). This indicates that contextual and structural protective factors will promote resilience when faced with challenges. The findings of this review indicate that personal traits, such as inner strength and hope, create resilience and that these personal traits are influenced by cultural and societal factors in the host country. However, cultural resilience is difficult to define since it can be linked to individual, contextual and structural resilience. Therefore, knowledge of the IEN's cultural background is crucial to the facilitation of a good integration process in the host country. We only found one study that describes both native nurses and the migrant nurse population (Schilgen et al., 2019). These authors conclude that migrant and native nurses share similar coping strategies in mastering occupational burdens, but that migrant nurses from different origins will perceive and emphasize their status as a community by sharing their commonalities. On the one hand, coping strategies are common to all nurses, which is in line with other literature (Stacey & Cook, 2019). On the other hand, family and being affiliated to friends from the same origins seem more prominent for IENs. This sense of community is especially evident in cultures and societies that hold a collectivistic perspective. In these cultures, ‘we’ are more important than ‘I’, in contrast to individualistic societies (Hofstede et al., 2011), and this seems central to the IENs’ ability to be resilient. The pride of being a nurse is possibly greater for IENs than for native nurses, and thus affects their experience of resilience. The reported studies suggest that IENs become stronger and more resilient as they face the challenges that follow migration, since they develop the ability and resources to navigate and negotiate in a meaningful way when confronted with challenges in the host country (Ungar, 2014). In summary, we might conclude that, as a driving force, the motivation to migrate is maintained in IENs by their belief in a good life in the host country, by their affiliation with family and friends and by their ability to adapt and cope with the challenges.

### Being resilient for better or for worse

A supportive environment, as described in the included studies, is in line with Ungar's study from 2007 (Ungar, 2014) describing supportive factors from studies conducted in 11 different countries. Seven protective factors were identified: relationships, a powerful sense of identity, personal control and efficacy, social justice, access to material resources, sense of cohesion and cultural adherence (Ungar, 2014). All of these protective factors are in line with the results of this review, except for social justice, which seems to have lacked attention over the chosen time period. This might be explained by the fact either that social justice was not addressed in the studies or that the participants were not concerned about social justice. However, it is more likely that the IENs were concerned about social justice, since many of them derived from countries where there was instability, violence and crime (Pung & Goh, 2017). It seems conceivable that the IENs’ very strength and ability to adapt and adjust to the new culture might affect the migrant nurse negatively, affecting both the credentialing process and the workplace. If nurses allow themselves to be exploited, and if they do not demand better working conditions, it could result in injustice rather than social justice for the nurses. Submissiveness that appears as adjustment and adaption can lead to exploitation, undermining requirements for better pay and improved working conditions for nurses in general, and accordingly creating a distance between the IENs and the native nurses. Therefore, structural resilience should be viewed with a critical eye. Trade unions and different nursing associations play an important role in preventing this possible distancing (Calenda & Bellini, 2021). In summary, we, therefore, realize that IENs’ resilience is crucial—for better or for worse—by supporting the protective factors that contribute to the IENs’ mastering their new life in the host country, while also recognizing that structural resilience might overshadow the requirements for equity and equality.

### Strengths and limitations

Almost all the articles reviewed in this literature review described challenges relating to language, culture, the credentialing process and separation from family. To cope with these challenges, individual, contextual and structural protective factors are described with an emphasis on the individual factors, and in line with Ungar's criticism of an often‐one‐sided understanding of resilience. This study is a clear indication that the discussion around equity and the integration of IENs must include the protective factors and the challenges faced by the IENs in order to ensure the best possible health service, where excellent nursing is provided by both native nurses and IENs. We have not been able to compare data regarding sex and gender as most studies included women, and there is a systematic lack of studies focusing on male IENs across the chosen time period.

To ensure study reliability, the articles were analysed by the research team in an interactive process with frequents meetings and discussion among the review authors which helped the reviewers to discover new perspectives and find similarities. Finally, the current review was performed in accordance with the PRISMA guidelines, and prospectively registered Prospero (Supplementary Table S1). The search strategies, the screening process and approaches for data analyses are clearly described in the method section and corresponding tables, with specific references to all approaches or processes used.

## IMPLICATION FOR NURSING AND HEALTH POLICY

The literature review indicates that IENs are motivated to remain in the host country despite individual and structural challenges, and findings suggest that supportive coping strategies, and a supportive environment entails resilience. This review is relevant to clinical practice and has implication for nursing and health policy because it aims for greater equity for the inclusion of both the knowledge of resilience and the IENs’ challenges. This knowledge might prevent nurses from being exploited and may subsequently improve outcomes for patients by improving the utilization of IEN resources. Authorities, managers in clinical practice, nursing educators, unions, nursing associations and nurses in general should be aware of all the protective factors that promote resilience, but also of the challenges that inhibit the resilience of IENs’ in their practice and social interactions.

## FUNDING INFORMATION

Oslo Metropolitan University funded this publication.

## AUTHOR CONTRIBUTIONS

Study design: KD, AKB; data collection: KD, AKB, LN, JS; data analyses: KD, AKB, LN, JS; study supervision: AKB; manuscript writing: KD, AKB; critical revisions for important intellectual content: KD, AKB, LN, JS.

## CONFLICT OF INTERESTS

The authors confirm that there are no conflicts of interest in this study regarding authorship contributions and permission to use, modify or translate scales in the researccess.

## Supporting information

Supplementary Table 1

Supplementary Table 2

Supplementary Table 3
