# Supplementary material for: Internationally educated nurses and resilience: A systematic literature review
Source: Int Nurs Rev. 2022 Jul 22;69(3):405–15. doi: 10.1111/inr.12787 (PMC9545834; doi:10.1111/inr.12787)
Supplement: Supplementary file 2 — Supplementary Table 2 [file INR-69-405-s002.docx]

# Supplemental table 2, Summary Table

| **Author, Year, Country, Title** | **Aim of study** | **Research design and methodology** | **Country of origin and working context, number, sex and age.** | **Description related to resilience** | **Concepts related to resilience** | **Challenges** |
| --- | --- | --- | --- | --- | --- | --- |
| Al-Hamdan et al (2015) , UK.  **Title:** Experiencing transformation: the case of Jordanian nurse immigrating to the UK. | How Jordanian nurses experienced the transition from home to host country to illuminate the elements of transformation. | Individual interviews. Content analysis. | Jordanian migrant nurses **in** UK,  20 males, 5 females, 11 <30 years, 13: 31-40 years, 1>40 years. | Professional transformation, personal transformation and sociocultural experiences.  No clear definition of resilience, focusing on transformation. | Individual coping strategies  Support from the family  Support from the workplace | Language barriers |
| Allen, (2018), USA. **Title:** Experiences of internationally educated nurses holding management positions in the United States: Descriptive phenomenological study. | To explore the experiences of internationally educated nurses in management positions in the US health care organizations to understand the obstacles and support these individuals experience when pursuing and working in managerial roles. | Phenomenological design. Individual interviews. | Nurses from the Philippines 2, India 3, China 1, Jamaica 1 **in** USA  5 females. 2 males, range 30-65. | 1.Supervisors in IENs acceptance; 2. Job challenges/responsibilities; 3. Cultural differences; 4. Language and communication; 5. Work relationships and support; and 6. Educational opportunities.  The participants were able to overcome the challenges through the support of organizational leaders and the desire to further their education and skills. Volunteering to serve on committees provided opportunities to expand their knowledge and skills, as well as to network with individuals in other areas of the organization. Some participants perceived that actively participating in committees contributed to their success. | Hope dreams and strength  Support from the workplace | Language barriers |
| Adhikari, (2013), UK.  **Title:** Empowered Wives and Frustrated Husbands: Nursing, Gender and Migrant Nepali in the UK. | This article illustrates how migrant nurses and their husbands have to accept a compromised social position, from being family breadwinners in Nepal to dependent husbands in the UK | Ethnographic approach., Individual interviews, participant observation and focus group discussions. | Over 100 nursing students, senior nurse managers, campus chiefs and nurse teachers, brokering agents, over 100 Nepali migrant nurses working **in** different parts of the UK, husbands and other family members.  242 female nurses, age not reported. Home country: Nepal | Women and nursing in contemporary Nepal, Nursing and migration: Increased family ijjet (honour) in modern Nepal, International nurse migration: Changing family and gender dynamics.  To empower or prepare women to work outside their domestic sphere and become  economically independent  Women s mobility and safety outside of the family home  A source of family pride | Hope dreams and strength  Support from the family | Cultural challenges |
| Alonso-Garbayo &Maben (2009), UK.  **Title:** Internationally recruited nurses from India and the Philippines in the United Kingdom: the decision to emigrate. | Examining factors in addition to the economic and professional aspects involved in this important decision of those of a social and cultural nature. | Case study. Individual interviews. | Nurses from India 6 and 15 the Philippines **in** UK  21 nurses and 10 managers, sex and age not described. | Three areas arising from the analysis comprised reasons for migration of an individual, social and cultural nature.  The importance of family and friends  cultural environment  To improve their economic situation,  professional, social or more personal factors | Individual coping strategies  Hope dreams and strength  Support from the family  Support from the workplace | Cultural challenges |
| Alexis & Shillingford, (2012), UK.  **Title:** Exploring the perceptions and work experiences of internationally recruited neonatal nurses: a qualitative study. | The aim of the study was to explore the experiences of internationally recruited neonatal nurses in the NHS in the UK. | Phenomenological approach. Individual interviews. | Nurses from Jamaica or the Philippines **in** UK  13 female, range 24-55. | The support mechanisms,  Unfamiliarity with family centered care,  Feelings of being treated like a child Coping strategies  As coping strategies: they needed to prove that they could cope despite being treated differently.  Individuals who feel valued and empowered | Hope dreams and strength | Separation from family |
| Bland & Woodbridge, (2011), New Zealand.  **Title:** From India to New Zealand--a challenging but rewarding passage. | This study, sought to explore the experiences of Indian RNs who, after completing a New Zealand RNBN programme in India, worked in New Zealand as RNs | Participatory action research. Focus groups. | Indian RNs who, after completing a New Zealand RNBN programme in India, worked **in** New Zealand as RNs.  7 women, 3 men, Age range 23-35. Home country: India | 1.To find something better for themselves - a desire to improve their nursing knowledge and skills,  2. Better employment opportunities. Working in a foreign country was expected to lead to better working conditions, greater access to health-related technology and higher remuneration. This particular group of nurses were positive their dream of a better future was on the way to being realised: it was a dream of one or two years, it was a dream of a whole future, so it is still progressing, and so far it is good. | Individual coping strategies  Hope dreams and strength  Support from society | Cultural challenges  A new way of working |
| Choi, Cook & Brunton (2019), New Zealand.  **Title:** Power distance and migrant nurses: The liminality of acculturation. | To explore the transitional discomforts experienced by IQNs, and to identify possible organizational and collegial steps that may ameliorate these challenges | Phenomenological approach. Individual interviews. | 5 Indian; 3 Filipina nurses **in** New Zealand  4 female and 4 male, range 28-34. | Un/learning and the hidden curriculum; Destabilisation of expertise; Preceptors and leaders as navigators; Finding one’s voice Enthusiastic about the relative egalitarianism they experienced,  Enjoying a newfound level of autonomy, and equity with medical colleagues | Support from society | Language barriers |
| Chun Tie, & Francis (2019), Australia. **Title:** Playing the game: A grounded theory of the integration of international nurses. | To explore how international nurses and Australian nurses adapt to work together in the Australian healthcare system and to develop a theory that explains this process. | Grounded theory. Online-survey, individual interviews and focus groups. | Home country of migrant nurses not reported **in** Australia  217 Australian qualified registered nurses and International qualified international nurses  88% female, age not reported, | (i)Joining the game;(ii) Learning the game, (iii)Playing by the rules, and (iv)The end game.  Adaptation: how local RNs and international RNs adapt to work cohesively together in the Australian health care system | Individual coping strategies  Support from the workplace  Support from society | Language barriers |
| Connor, (2016), USA.  **Title:** Cultural Influence on Coping Strategies of Filipino Immigrant Nurses. | Discusses the strategies that Filipino IENs  use to cope effectively with their work-related and nonwork-related stress explored stress and coping in a sample of  A part of a larger study: Filipino IENs within  the context of the immigration and adaptation process | Cross-sectional descriptive  design. Individual interviews. | Nurses from the Philippines **in** USA  20 female, range 28-48. | (a) familial coping, (b) intracultural coping, (c) fate and faith-based coping, (d) forbearance (patience and self-control) and contentment, (e) affirming the nursing profession and proving themselves, and (f) escape and avoidance  Coping is defined as the individuals cognitive and behavioral responses to manage the internal and external demands of stress (Folkman, 2013; Lazarus & Folkman, 1987) | Individual coping strategies  Hope dreams and strength  Support from the family  Support from the workplace | Cultural challenges  Stress |
| Dahl, et al. (2017), Norway.  **Title**: Conscientious and proud but challenged as a stranger: Immigrant nurses' perceptions and descriptions of the Norwegian healthcare system. | To explore how immigrant nurses, all educated as nurses in their home countries, experience working as a nurse in Norway. | Social constructivism Survey, open-ended questions. | 144 Immigrants nurses from 18 different countries outside the EU. Countries in Asia and Eastern Europe were predominant **in** Norway  84% women and 16% men, age mean 32. | Conscientious assisting and proud as nurse Impressed but challenged as strangers.  Resilience as cultural competence which includes cultural awareness, cultural knowledge, cultural skill, cultural encounter and cultural sensitivity | Individual coping strategies  Hope dreams and strength | Language barriers  Cultural challenges |
| Eriksson, et.al  (2018), Sweden. **Title:** Internationally educated nurses' and medical graduates' experiences of getting a license and practicing in Sweden - a qualitative interview study. | To describe IENs and IMGs experiences of getting a license to practice and work in the Swedish health and social care system. To evaluate their ability to use their intercultural competence at work, and whether intercultural competence could be an asset in an increasingly multicultural society. | Descriptive design. Individual interviews. | Nurses from Bosnia, Bulgaria, Germany, Great Britain,  New Zealand, Poland, Serbia, Spain, Sudan, Italy, Syria, Romania, Greece, Uzbekistan **in** Sweden  13 female and 9 male, range 35-39 | Getting a license -a different story, The work is familiar, yet a lot is new, Trying to master a new language.  Definition of intercultural competence: one ability to communicate effectively and appropriately in intercultural situations based on one intercultural knowledge, skills, and attitudes | Support from society | Language barriers  Inefficient credentialing process |
| Eriksson & Engstøm, (2018), Sweden.  **Title:** Internationally educated nurses' descriptions of their access to structural empowerment while working in another country's health care context. | To examine internationally educated nurses’ experiences of empowerment structures using Kanters theory of structural empowerment as a framework | Descriptive design. Individual interviews. | Nurses from Algeria, Bosnia, Bulgaria, Germany, Great Britain, New Zealand, Poland, Serbia, Spain, Sudan **in** Sweden  1 male ,10 female, age 25-59 (35). | Access to information, support, resources, opportunities, formal power and to informal power.  Empowering structures described in Kanter theory” such as support, information, and informal power may help IENs adjust to the new work environment. | Hope dreams and strength  Support from the workplace  Support from society | Language barriers |
| Fong et.al (2005), Singapore. **Title**: The expectations and experiences of Myanmar nursing aides working in an inpatient hospice in Singapore. | Explore the experience of Myanmar nurses working as nursing aides in the palliative care setting | Exploratory research. Focus groups. | 18 Myanmar nurses, working as nursing aids in an inpatient hospice **in** Singapore  sex and age not reported | Nursing aids attitude toward life threatening illness stressors coping strategies | Individual coping strategies | Coping stressors |
| Healee &Inada (2016), New Zealand.  **Title:** Working with difference: Thematic concepts of Japanese nurses working in New Zealand. | The purpose of this study was to compare the differences experienced by Japanese nurses working in New Zealand from an organizational and personal perspective | Descriptive design. Individual interviews. | Japanese nurses **in** New Zealand  9 female, Age range 30-40, Home country: Japan | Finding a voice Two subthemes: accommodating difference and learning to speak up Finding a voice was a method of negotiating how they practised nursing in New Zealand compared to Japan.  To learn to accommodate difference while learning to speak up | Individual coping strategies  Hope dreams and strength  Support from society | Cultural challenges |
| Iheduru-Anderson &Wahi, (2018), USA.  **Title:** Experiences of Nigerian Internationally Educated Nurses Transitioning to United States Health Care Settings. | To characterize the facilitators and barriers to transition of Nigerian IENs (NIENs) to the United States health care setting | Phenomenological approach. Individual interviews. | Nigerian nurses **in** USA  6 females mean 39 years | Fear/anger and disappointment (FAD), Road/journey to success/overcoming challenges (RJO),  Moving forward (MF) Road/journey to success/overcoming challenges:  The subtheme of resilience and not giving up includes gaining assertiveness as well as acquiring culturally acceptable behaviors expected in the United States workplace. Empowerment that comes with learning the system and becoming better acquainted with how to live and work in USA | Individual coping strategies  Hope dreams and strength  Support from the workplace | Social adjustment |
| Jenkins, & Huntington (2016), New Zealand.  **Title:** "We are the international nurses “: An exploration of internationally qualified nurses' experiences of transitioning to New Zealand and working in aged care. | To explore the experiences of Filipino and Indian IQNs who transitioned to New Zealand as RNs in aged care. | Exploratory study. Individual interviews and focus groups. | Nurses from India and the Philippines **in** New Zealand  5 Female and 1 male, Age 27-31, mean: 29, | The physical transition, the social  transition and the professional transition  Strategies for coping with the difficulties encountered | Individual coping strategies  Support from the workplace | Inefficient credentialing process  Separation from family |
| Jose, (2011), USA. **Title**: Lived experiences of internationally educated nurses in hospitals in the United States of America. | To elicit and describe the lived experiences of internationally educated nurses (IENs) who work in a multi-hospital medical centre in the urban USA. | Phenomenology. Individual interviews. | Nurses from the Philippines (8), India (7) Nigeria (5) **in** USA  3 Male and 17 females, Participants ranged in age from 23 to 44 years with an average of 33.2 years. | (1) dreams of a better life,  (2) a difficult journey,  (3) a shocking reality,  (4) rising above the challenges,  (5) feeling and doing better (6) ready to help | Hope dreams and strength | Language barriers  Cultural challenges |
| Jose, (2009), USA. **Title**: A phenomenological study of the lived experiences of foreign educated nurses working in the United States of America. | To explore and describe the lived experiences of foreign educated nurses (FENs) working in the United States of America. Describe strategies the FENs reveal they used to adapt and cope with challenges. | Phenomenological design. Individual interviews. | Nurses from the Philippines (8), India (7), and Nigeria (5) **in** USA  Male 3 and Female 17, range 23-44 | Dreams of a better life Difficulties of the journey A shocking reality Rising above the challenges Feeling and doing better Ready to help others | Individual coping strategies  Hope dreams and strength  Support from the workplace  Support from society | Separation from family |
| Kishi et.al.,(2014), Australia.  **Title:** A Model of Adaptation of Overseas Nurses: Exploring the Experiences of Japanese Nurses Working in Australia. | To investigate the experiences of Japanese nurses and their adaptation to their work environment in Australia. | Individual interviews. | 14 Japanese nurses **in** Australia  Female: 13, Male: 1, range 30-59. | Seeking, acclimatizing and settling  Adaptation: their fulfilment and develop their capacity, which is essential for reaching self-actualization or fulfilment | Individual coping strategies  Support from the workplace | Language barriers |
| Liou & Cheng , (2011), USA.  **Title:** Experiences of a Taiwanese nurse in the United States. | To explore and interpret the lived experience of a Taiwanese nurse working in a U.S. hospital | Hermeneutic, phenomenology and case study approach. Individual interviews. | One nurse from Taiwan **in** USA  1 female. 35 years. | (a) frustration in language and communication; (b) cultural differences in patient care; (c) support from work environment; and (d) advantages of working in the U.S. nursing system. support from many of her nurse colleagues  and some patients. This support helped ease her hurt feelings and gave her energy to move forward in her job.  Human rights, patient -nurse ratio | Support from the workplace | Language barriers |
| Lin, (2014), USA. **Title**: Filipina nurses' transition into the US hospital system. | Exploring how Filipina nurses’ transition into their role as nurses and adapt to nursing practice in the US. | Individual interviews. | Nurses from the Philippines **in** USA  31 females, age not reported | Conceptualizing US nursing, reacting and interacting. Focus on transition from one culture to another, to ease the transition process: Seek help from others, learn to face/deal with it, Seek spiritual support, Seek support from others, Mingle with other Filipinos, Maintain open-minded attitudes, Learn from Filipino mentors, Embrace the US culture, Learn to speak up/be assertive | Individual coping strategies  Support from the workplace  Support from society | Language barriers  Cultural challenges  Inefficient credentialing process |
| Magnusdottir, (2005), Iceland. **Title:** Overcoming strangeness and communication barriers: a phenomenological study of becoming a foreign nurse. | To generate an understanding of the foreign nurses’ experience with the purpose of contributing to a constructive international nursing/health care climate in Iceland. | Phenomenology, hermeneutic and constructivism. Individual interviews. | Nurses from 7 western and 4 non-western countries **in** Iceland  11, sex not described, age range 20-35. | First theme: tackling the initial, multiple challenges Second theme: becoming an outsider and the need to be let in Third theme: struggling with the language barrier Fourth theme: adjusting to a different work culture Fifth theme: overcoming challenges to win through  Nearly all felt they had grown through the experience. They spoke about being stronger, more independent and having better self-knowledge. Some felt they were more open-minded, more expressive and softer persons. | Support from the workplace | Language barriers |
| Philip et.al.,(2019), Australia.  **Title**: Overseas Qualified Nurses' (OQNs) perspectives and experiences of intraprofessional and nurse-patient communication through a Community of Practice lens. | To explore the barriers and enablers of clinical communication experiences of OQNs from their perspective using a Communities of Practice framework | Individual interviews. | Nurses from the Philippines 10, India 8, Singapore 1, Africa 1 **in** Australia  female 17, male 3, age range 25 to 50 | Internal factors relating to self and external factors relating to interactions with members of the CoP  Willingness to adapt and learn. An engagement of the self to the broader community  Adjustment for smooth transition and progression through their nursing career | Individual coping strategies | Language barriers |
| Ramji &St.Pierre, (2018,) Canada. **Title**: Unpacking "two-way" workplace integration of internationally educated nurses. | Understanding workplace integration from perspectives of both IENs and   other stakeholders. | Mixed Methods, Instrumental case study approach. Documents review,   individual interviews,   survey, and ~~focus~~ groups | Home country not reported  28 and 50 % were IEN in Canada, age not reported,  Fifty percent were IENs and the rest of the participants included peers/mentors (18%), managers/directors (21%) and senior leaders (11%) **in** Canada. | (1)  Respecting diversity and difference,   (2) Adopting inclusive practices,   (3). Striving to achieve equity. | Support from the workplace | Language barriers  Cultural challenges |
| Rodriguez &Hoga ( 2014), Chile.  **Title**: Cultural experiences of immigrant nurses at two hospitals in Chile. | To explore the cultural experiences of nurses who immigrated to Chile. | Ethnography. Observation and interviews. | Migrant nurses from graduated **in** Brazil (1), Colombia (7), Ecuador (3) and Uruguay (4) in Chile.  15 female, Age range 24-44. | In search of better horizons the decision to immigrate to Chile; Gaining trust and establishing a support network employability and professional performance; Seeking peoples acceptance professional adaptation in a new cultural scenario  Professional development aspirations  Better living conditions and quality of life  Help from family members, friends, boyfriends or husbands  The main facilities involved the existence of  job opportunities, some similarities in the professional  education and support from patients. | Support from the family | Language barriers  Social adjustment |
| Ronquillo, (2012), Canada.  **Title:** Leaving the Philippines: oral histories of nurses' transition to Canadian nursing practice. | This study examines the transition experiences of Filipino nurses who immigrated to Canada between 1970 and 2000 | Individual interviews. | 9 Filipino nurse immigrants residing **in** the provinces of Alberta and British Columbia  Age from early thirties to late fifties. | Family First, Nursing Later Nursing in Canada: Different Expectations Being Foreign: Proving Oneself and Perceptions of Discrimination  Immigration journey, transition experiences  Familial responsibility and sacrifice, shifting expectations of what it means to be a nurse in Canada, and perceptions of feeling foreign and the need to prove oneself were common themes. | Support from the family | Social adjustment  Separation from family |
| Salami &Covell (2018) , Canada. **Title:** Downward occupational mobility of baccalaureate-prepared, internationally educated nurses to licensed practical nurses. | The experience of baccalaureate-prepared, internationally educated nurses who work as licensed practical nurses in Canada | Exploratory design. Individual interviews. | Nurses from the Philippines (N= 9), India (N=3), Nigeria (N=1), Mauritius (N=1) working as an LPN **in** Canada.  12 females, 2 males, range 27-52 | Hope for a better personal and professional life.  Barriers to workforce integration as registered nurses and discover an easier path in the licensed practical nurse registration process;  Deskilling and ambivalent skill recognition;  Dissatisfied as a licensed practical nurse in Canada. | Hope dreams and strength | Inefficient credentialing process |
| Salma &Ogilvie, (2012), Canada. **Title**: Career advancement and educational opportunities: experiences and perceptions of internationally educated nurses. | To encourage ethical IEN employment by understanding their needs, and (b)  To maintain successful IEN recruitment by understanding factors which increase retention | Interpretive descriptive methodology. Individual interviews. | Registered IEN nurses from Guyana, China, Iran, Philippines, India, Britain, and New Zealand **in** Canada  1 male and 10 females, two participants were between 20 and 30 years of age.  Three participants were between 30 and 40 years of age, eight participants were above 45years of age | Being a mother for my children  seeking financial stability,  Pride  Need for financial support  Skill recognition  Personal responsibility, resilience, and personal motivation for success. | Hope dreams and strength | A new way of working |
| Schilgen & Mosgo (2019), Germany.  **Title:** Work-related barriers and resources of migrant and autochthonous homecare nurses in Germany: A qualitative comparative study. | To explore migrant and minority homecare nurses’ psychosocial strains and stressors, resources and coping strategies | Phenomenological approach. Individual interviews. | Nurses from Afghanistan, Ghana, Africa, Bosnia, Brazil, Colombia, Croatia, Ecuador, Finland, Indonesia, Latvia, Russia, Uganda, Yugoslavia, Iran, Turkey and Germany **in** Germany  24 native nurses and 24 migrant nurses, Migrant: 21 f and 3 m  Native :20 f and 4 m Age: Migrant:23-68(42.92) Native:30-62 (45.17). | The three leading subjects of the study were barriers resources and coping strategies. These three subjects were then embedded in four contexts, namely work in general colleagues’ direct supervisor and clients. Within the contexts of colleagues and clients, a further subdivision into intercultural and general fallowed the distinct description of cultural influences on the work of the nurses under study. Thus, one subject and its specific context formed a cluster | Support from the workplace | Language barriers  Social adjustment |
| Sochan, &Singh (2007)Canada.  **Title**: Acculturation and socialization: voices of internationally educated nurses in Ontario. | This study explored the experiences of IENs in their journey to become licensed RNs in Ontario. A number of questions guided this exploration. What are their personal stories? Are their experiences similar? Is there atypical journey to become an RN in Ontario? | Biographical Narrative methodology. Individual interviews. | Nurses from the Philippines (5), Mainland China (2), India (2), South Korea (2) and Ukraine (1).**in** Canada  1 male and 11 females, age not reported | The Told portion of this overall theme, including: (a) wanting the Canadian dream (of becoming an Ontario RN); (b) discovering that their home-country nursing qualification does not meet Ontario entry to practice; and (c) the redefined Canadian dream of returning to nursing school to upgrade their nursing qualifications.  Untold interpretations of these three themes are: (a)hope, (b) disillusionment, and (c) navigating disillusionment. accept their fate and have reconciled themselves to returning to school to upgrade their nursing qualifications to meet Ontario entry to practice requirements.  Viewed this journey with an  ironical sense of humour. | Individual coping strategies  Hope dreams and strength | Language barriers  Cultural challenges  Inefficient credentialing process  Separation from family |
| Stubbs, (2017), UK. **Title:** Recruitment of nurses from India and their experiences of an Overseas Nurses Program. | To explore the transition experiences of nurses recruited from India to London to work in critical care settings in 2011. | A descriptive approach. Individual interviews. | Nurses from India, **in** UK  11 females and 5 males, age range 25-33. | Autonomy and responsibility,  Language,  Culture (food and climate),  Loneliness and work challenges | Support from society | Language barriers |
| Vafeas & Hendrix , (2017), Australia. **Title**: A heuristic study of UK nurses` migration to WA: Living the dream downunder. | To understand the experience of migration for RNs moving from the UK to WA | Heuristic inquiry. Focus group, individual interviews, and a journal (??). | UK nurses **in** Australia  18 females and 3 males, range 26-51. | developing resilience finding a new professional identity having the ability to adapt to a new life Coping strategy. These qualities incorporated flexibility being able to establish a sense of new personal and professional self-identity; and being able to adjust to change. | Individual coping strategies | Cultural challenges |
| Walters, (2008), Australia.  **Title**: The experiences, challenges and rewards of nurses from South Asia in the process of entering the Australian nursing system. | To explore, identify and document, in the words and expressions of participants, the lived experience of immigration, living and working as a nurse in a foreign country. | Narrative analysis. Individual interviews. | 16 south Asian nurses **in** Australia  14 females and 2 males, range 26-41 | Trust and fear, English language requirements, Immigration, Belonging, integration and family, Living and working in the West | Hope dreams and strength  Support from society | Cultural challenges  Inefficient credentialing process |
| Winkelmann-Gleed & Seeley ( 2005), UK  **Title**: International nursing. Strangers in a British World? Integration of international nurses. | To examines the experiences of recently internationally qualified migrant nurses to Britain and explores their stories with the aim of understanding aspects of their work-related identities | Mixed Method. Survey and individual (?) interviews. | Internationally qualified, foreign-born nurses from Asian, White, Black or Black British **in** London UK.  20 females and 2 males  S:115 females and 25 men, mean 34,19 years | The perception of migrants by British-trained nurses and patients: hardship Migrant nurses" integration; Carer progression  Experienced fair and respectful interaction | Support from the workplace  Support from society | A new way of working  Social adjustment |
| Wheeler &Hepburn (2014), USA.  **Title:** The experience of discrimination by US and Internationally educated nurses in hospital practice in the USA: a qualitative study. | To document experiences of nurses educated abroad and in the USA in 2 urban hospitals in the southeastern USA. | Explorative approach using structuration theory(??). Individual interviews. | Nurses from Caribbean, African American, European Union,  Sub-Saharan Africa, Southwest Asia, East Asia,  Pacific, Oceania **in** USA  1. interview :42 IEN and 40 USA educated nurses, all female  2.interview :22 IEN and 18 USRN, all female, US RNs: 39, IENs: 45 | Discrimination by patients, Discrimination by supervisory hospital personnel, Discrimination by fellow nurses, Coping.  Rely on personal values to help them ignore it,  Excuse the behaviour,  Confront it,  Change units or shifts or  Leave their positions and/or they work harder to prove themselves to everyone around them | Individual coping strategies  Hope dreams and strength | Social adjustment |
| Wolcott & Mace (2013), USA.  **Title**: Integration of internationally educated nurses into the U.S. Workforce. | To explore the experiences of internationally educated nurses and the nurse managers and educators working with them, to understand the issues, and to highlight potential solutions for addressing integration challenges. | Grounded theory. Individual interviews. | 5 IEN., 4 Educators and 4 Managers from Denmark, Germany, India, Philippines, and Portugal **in** USA  sex and age not reported  . | Communication difficulties; financial challenges; the need for outside social support; and educational orientations focused on culture, nurse role, and communication techniques.  Roy defined adaptive as the human’s capacity to adjust effectively to changes in the environment and affect the environment. | Hope dreams and strength | Language barriers  Inefficient credentialing process |
| Xu, &Kim (2008), USA.  **Title:** Adaptation and transformation through (un)learning: lived experiences of immigrant Chinese nurses in US healthcare environment. | This study examines the lived experiences of a group of Chinese nurses working in the US healthcare environment. | Phenomenology. Individual interviews. | 9 female nurses from China **in** USA  Mean 40,4 (32-51). | (a)Communication as the most daunting challenge, especially during initial transition of their first job; (b) different and even conflicting professional values and roles/expectations of the nurse between the United States and China; (c) marginalization, inequality, and discrimination;(d) transformation through clinging to hope, (un)learning, and resilience; and (e) cultural dissonance  Enjoyed their work, and pursued their career dreams trough self-confidence, strength, assertiveness, persistence, and determination; valuing education and life-long learning; taking initiative; never giving up. | Hope dreams and strength | Language barriers  Social adjustment |
