# Supplementary material for: Internationally educated nurses and resilience: A systematic literature review
Source: Int Nurs Rev. 2022 Jul 22;69(3):405–15. doi: 10.1111/inr.12787 (PMC9545834; doi:10.1111/inr.12787)
Supplement: Supplementary file 3 — Supplementary Table 3 [file INR-69-405-s001.docx]

**Supplemental table 3: The Joanna Briggs' Checklist for Qualitative Research Studies**

| **Author, Year** | **1. Is there congruity between the stated philosophical perspective and the research methodology?** | **2. Is there congruity between the research methodology and the research question or objectives?** | **3. Is there congruity between the research methodology and the methods used to collect data?** | **4. Is there congruity between the research methodology and the representation and analysis of data?** | **5. Is there congruity between the research methodology and the interpretation of results?** | **6. Is there a statement locating the researcher culturally or theoretically?** | **7. Is the influence of the researcher on the research, and vice- versa, addressed?** | **8. Are participants, and their voices, adequately represented?** | **9. Is the research ethical according to current criteria or, for recent studies, and is there evidence of ethical approval by an appropriate body?** | **10. Do the conclusions drawn in the research report flow from the analysis, or interpretation, of the data?** |
| --- | --- | --- | --- | --- | --- | --- | --- | --- | --- | --- |
| Adhikari, 2013 | Yes | Unclear | Yes | Yes | Yes | No | No | Yes | Unclear | Unclear |
| Alexis, 2012 | Yes | Yes | Yes | Yes | Yes | Unclear | Yes |  | Yes | Yes |
| Allen, 2018 | Yes | Yes | Yes | Yes | Yes | Yes | Unclear | Unclear | Yes | Yes |
| Al-Hamdan, 2015 | Unclear | Yes | Unclear | Unclear | Unclear | No | No | Unclear | Yes | Yes |
| Alonso-Garbayo, 2009 | Yes | Unclear | Unclear | Yes | Yes | Yes | Yes | Unclear | Yes | Yes |
| Bland, 2011 | Yes | Yes | Unclear | Unclear | Unclear | Yes | Unclear | Unclear | Yes | Unclear |
| Choi, 2019 | Yes | Yes | Unclear | Unclear | Yes | Yes | Unclear | Yes | Yes | Yes |
| ChunTie, 2019 | Yes | Unclear | Unclear | Unclear | Yes | No | Unclear | Unclear | Yes | Yes |
| Connor, 2016 | Unclear | Yes | Yes | Yes | Yes | Yes | Unclear | Yes | Yes | Yes |
| Dahl, 2017 | Yes | Yes | Yes | Yes | Yes | Unclear | Yes | Yes | Yes | Yes |
| Eriksson, 2018 | Yes | Yes | Yes | Yes | Yes | No | No | Yes | Yes | Yes |
| Eriksson, 2018 | Yes | Yes | Yes | Yes | Yes | Unclear | Unclear | Yes | Yes | Yes |
| Fong, 2005 | Unclear | Yes | Yes | Yes | Yes | Unclear | Unclear | Unclear | Yes | Unclear |
| Iheduru-Anderson, 2018 | Yes | Yes | Unclear | Yes | Yes | Unclear | Unclear | Yes | Yes | Yes |
| Jenkins, 2016 | Unclear | Yes | Yes | Yes | Yes | Unclear | Unclear | Yes | Yes | Yes |
| Jose, 2011 | Yes | Yes | Yes | Yes | Yes | Unclear | Yes | Yes | Yes | Yes |
| Jose, 2009 | Yes | Yes | Yes | Yes | Yes | Unclear | Unclear | Yes | Yes | Yes |
| Kishi, 2014 | Yes | Yes | Yes | Yes | Yes | Unclear | No | Yes | Yes | Yes |
| Lin, 2014 | Unclear | Yes | Yes | Unclear | Yes | No | Unclear | Unclear | Yes | Yes |
| Liou, 2011 | Yes | Unclear | Unclear | Yes | Yes | Unclear | Unclear | Yes | Unclear | Yes |
| Magnusdottir, 2005 | Yes | Yes | Yes | Yes | Yes | Unclear | Unclear | Yes | Yes | Yes |
| Philip, 2019 | Unclear | Unclear | Yes | Yes | Yes | Yes | Yes | Unclear | Yes | Yes |
| Ramji, 2018 | Yes | Yes | Yes | Yes | Unclear | Unclear | Unclear | Yes | Yes | Yes |
| Rodriguez, 2014 | Yes | Yes | Unclear | Yes | Yes | Yes | No | Unclear | Yes | Yes |
| Ronquillo, 2012 | Yes | Yes | Unclear | Unclear | Yes | Yes | Yes | Unclear | Yes | Yes |
| Salami, 2018 | Yes | Yes | Unclear | Yes | Yes | Unclear | Unclear | Unclear | Yes | Yes |
| Salma, 2012 | Unclear | Yes | Unclear | Yes | Yes | Yes | Yes | Yes | Yes | Unclear |
| Schilgen, 2019 | Yes | Yes | Yes | Unclear | Yes | Unclear | Unclear | Yes | Yes | Yes |
| Sochan, 2007 | Yes | Yes | Yes | Yes | Yes | Yes | Unclear | Unclear | Yes | Yes |
| Stubbs, 2017 | Unclear | Yes | Yes | Yes | Yes | No | No | Yes | Yes | Yes |
| Healee, 2016 | Yes | Unclear | Yes | Yes | Yes | Unclear | Unclear | Yes | Yes | Yes |
| Vafeas, 2018 | Yes | Unclear | Unclear | Unclear | Yes | Unclear | Unclear | Yes | Yes | Yes |
| Walters, 2008 | Yes | Yes | Yes | Yes | Yes | No | No | Yes | Yes | Yes |
| Wheeler, 2014 | Yes | Yes | Yes | Yes | Yes | Yes | Yes | Yes | Yes | Yes |
| Winkelmann-Gleed, 2005 | Yes | Yes | Yes | Unclear | Unclear | Unclear | No | Yes | Yes | Unclear |
| Wolcott, 2013 | Yes | Yes | Yes | Yes | Yes | No | Unclear | Unclear | Yes | Yes |
| Xu, 2008 | Yes | Yes | Yes | Yes | Yes | Yes | Yes | Unclear | Yes | Yes |

| Author, Year, Country, Title | Aim of study | Research design and methodology | Method of recruitment of participants | Population description | Total number of participants, sex, age, and country of origin | Main themes | How is resilience defined? | Comments/Implications | |  |  |  |  |  |  |  |  |
| --- | --- | --- | --- | --- | --- | --- | --- | --- | --- | --- | --- | --- | --- | --- | --- | --- | --- |
| Alonso-Garbayo, 2009, UK. Title: Internationally recruited nurses from India and the Philippines in the United Kingdom: the decision to emigrate. | Examining factors in addition to the economic and professional aspects involved in this important decision â€“ those of a social and cultural nature. | a qualitative interpretative approach, Individual interview. Start in 2005, study conducted over 18 months | Clinic | Six Indian nurses, who were interviewed three times over eight months from the date of arrival in the UK in 2005, and 10 of their managers and mentors. The second element comprised Filipina nurses recruited from two cohorts; six nurses who had been in post in the United Kingdom for 18 months and nine nurses recruited by the Trust four years previously | 21 nurses and 10 managers, sex and age not described. Home country: India 6 and the Philippines 15 | Three areas arising from the analysis comprised reasons for migration of an individual, social and cultural nature. | The importance of family and friends  cultural environment To improve their economic situation, professional, social or more personal factors | This study shows the diverse motivations of nurses from different countries and with different migratory backgrounds and provides evidence that factors other than economic factors influence nurses' decision to emigrate. | | | | | | | | | |
| Adhikari, 2013, UK. Title: Empowered Wives and Frustrated Husbands: Nursing, Gender and Migrant Nepali in the UK. | This article illustrates how migrant nurses and their husbands haveto accept a compromised social position, from being family bread-winners in Nepal to depen-dent husbands in the UK | Qualitative ethnographic style, Combination of individual interviews, participant observation and focus group discussions. Start Jul-06, December 2008 and a following up 2009 | Other: Interview samples were gathered by snowballing techniques, using multiple sources to identify informants and to diversify the interview base. | Over 100 nursing students, senior nurse managers, campus chiefs and nurse teachers, brokering agents, over 100 Nepali migrant nurses in different parts of the UK, husbands and other family members. | 242 female nurses, age not reported. Home country: Nepal | Women and nursing in contemporary Nepal, Nursing and migration: Increased family ijjet (honour) in modern Nepal, International nurse migration: Changing family and gender dynamics. | To empower or prepare women to work outside their domestic sphere and become  economically independent  Women s mobility and safety outside of the family home A source of family pride | The Nepal government current women migration policy has created a serious controversy,which requires urgent policy attention. Because of British work permit regulations, Nepali nurses migrate to the UK on their own. Typically the UK government gives little consideration to how its international nurse recruitment practices and work permit policy affects migrants family life. There is a need for a family-friendly immigration policy. Female / nurse migration has a profound impact on nurses families lives in the UK. This area requires further inquiry. |  |  |  |  |  |  |  |  |  |
| ChunTie, 2019, Australia. Title: Playing the game: A grounded theory of the integration of international nurses. | To explore how international nurses and Australian nurses adapt to work together in the Australian healthcaresystem and to develop a theory that explains this process. | Grounded theory, Combination of online-survey, individual interviews and focus groups. Data collection period not clear | Other: A link to the anonymous survey was disseminated via Australian professional and industrial nursing organisations. Recruitment for interviews was based on anonymous comple-tion of the online survey and their subsequent willingness to be involved. | Australian qualified registered nurses and International qualified international nurses working in Australia. | 217, 88% female, age not reported, Home country not reported | (i)Joining the game;(ii)Learning the game,(iii)Playing by the rules ,and (iv)The end game. | Adaptation: how local RNs and international RNs adapt to work cohesively together in the Australian health care system | Regulators and organizations share responsibility for IQRN to have access to information and appropriate orientation. | | | | | | | | | |
| Schilgen, 2019, Germany. Tilte: Work-related barriers and resources of migrant and autochthonous homecare nurses in Germany: A qualitative comparative study. | To explore migrant and minority homecare nurses psychosocial strains and stressors, resources and coping strategies | Phenomenological research approach, Individual interview. Start 02-2017 to 01/07/2017 | Other: The service's nurses management via email  (top-down recruitment) | i) registered nurses and nursing assis-tants with German certificate or whose certificates are in a validation process,  (ii)born in Germany or born abroad and migrated to Germany; (iii) had been practicing in homecare for at least one year;  (iv) being employed on a full-time or part-time basis for at least one year in homecare or for six month  (v) at least 18 years old. | 24 native nurses and 24 migrant nurses, Migrant: 21 f and 3 m Native :20 f and 4 m Age: Migrant:23-68(42.92) Native:30-62 (45.17). Home country Afghanistan, Ghana, Africa, Bosnia, Brazil, Colombia, Croatia, Ecuador, Finland, Indonesia, Latvia, Russia, Uganda, Yugoslavia, Iran, Turkey and Germany | The three leading subjects of the study wereâ€œbarriersâ€,â€œresourcesâ€ andâ€œcoping strategiesâ€. These three subjects were then embedded in four contexts, namely â€œwork in generalâ€, â€œcolleaguesâ€,â€œdirect supervisorâ€ and â€œclientsâ€. Within the contexts of colleagues and clients, a further subdivision into â€œinterculturalâ€ andâ€œ general â€allowed the distinct description of cultural influences on the work of the nurses under study. Thus, one subject and its specific context formed a cluster | Nurses report that they converse with their colleagues about problems or issues that burden them. This exchange of views helps them to reflect the situation and to find a way to solve it or to accept the situation as it is. The personal exchange with clients and colleagues helps nurses to cope with burdening client-related situations. Feeling appreciated or receiving signs of gratitude motivate nurses to persevere in challenging times. The mutual support among colleagues, the direct verbal exchange with the supervisor at eye level and the perception of gratitude/appreciation are coping strategies that helps them to successfully master barriers occurring at their workplace. Migrant nurses of different origin perceive their status as migrants as a sense of community by sharing the same destinyâ€“this appears as an important resource for migrant and minority nurses. | Good collaboration in the team and a having an appreciative supervisor are resources that support migrant and minority nurses as well as autochthonous nurses to face the stressors and to cope with those | | | | | | | | | |
| Jenkins, 2016, New Zealand. Title: "WE ARE THE INTERNATIONAL NURSES": AN EXPLORATION OF INTERNATIONALLY QUALIFIED NURSES' EXPERIENCES OF TRANSITIONING TO NEW ZEALAND AND WORKING IN AGED CARE. | To explore the experiences of Filipino and Indian IQNs who transitioned to New Zealand as RNs in aged care. | Exploratory study, Combination of individual interviews and focus groups. Start Jul-14 to 01/09/2014 | Other: flyer distributed by an intermediary within the facility | Be an IQN from India or the Philippines,  have experienced the phenomenon of transitioning directly to New Zealand, have gained nursing registration as an RN in New Zealand, and be working in aged care  at the time of the study. | 6, 5 Female and 1 male, Age 27-31, mean: 29, Home coutry: India and the Philippines | The physical transition,the social  transition and the professional transition | Strategies for coping with the difficulties encountered | Findings from the physical, social, and professional transitions raise questions about how these nurses are supported during this time and provide valuable insights that should assist with future workforce planning, policy making, and research. | | | | | | | | | |
| Philip, 2019, Australia. Tilte: Overseas Qualified Nurses' (OQNs) perspectives and experiences of intraprofessional and nurse-patient communication through a Community of Practice lens. | To explore the barriers and enablers of clinical communication experiences of OQNs from their perspective using a Communities of Practice framework | Unclearly described, qualitative with an theoretical underpinning of the study, incorporating the Communities of Practice model, Individual interview. Start 02-16 to 01/03/2017 | Clinic | Completion of a nursing degree in countries where English is not the first language; have had practiced in the country of origin as a registered nurse; have current registration in Australia and have been practicing for at least six months, and working in acute and subacute clinical settings. | female 17, male 3, age range 25 to 50. Home counry: Phillippines 10, India 8, Singapore 1, Africa 1 | Internal factors relating to self and external factors relating to interactions with members of the CoP | Willingness to adapt and learn. An engagement of the self to the broader community Adjustment for smooth transition and progression through their nursing career | Enable OQN to be empowered for competent communication practice Provision of an ongoing supportive learning environment with opportunities for constructive feedback will help OQNs move from peripheral participation to full participation in health care encounters. |  |  |  |  |  |  |  |  |  |
| Ramji, 2018, Canada. Title: Unpacking "two-way" workplace integration of internationally educated nurses. | Understanding workplace integration from perspectives of both IENs and  other stakeholders. | Mixed Method, Instrumental case study approach. Documents review,  twenty-eight interviews,  socio-demographic survey, five focus groups involving IENs and other stakeholders. Start 10-14 to 01/03/2015 | Other: Presentations at meetings, information letters and posters. Snowball technique | ... participants was diverse with respect to age range, gender, country of origin and nursing education, ethnoracial heritage, immigration status and number of years and types of nursing or other professional work experiences. Fifty percent were IENs and the rest of the participants included peers/mentors (18%), managers/directors (21%) and senior leaders (11%). | 28 and 50 % were IEN, age not reported, Home country not reported | (1) Respecting diversity and difference,  (2) Adopting inclusive practices,  (3). Striving to achieve equity. | Respecting diversity and difference, adopting inclusive practice | The individual IEN and the organization levels, it is reasonable that facilitating workplace integration is a continuous work in progress. | | | | | | | | | |
| Dahl, 2017, Norway. Title: Conscientious and proud but challenged as a stranger: Immigrant nurses' perceptions and descriptions of the Norwegian healthcare system. | To explore how immigrant nurses, all educated as nurses in their home countries, experience working as a nurse in Norway. | Social constructivism as qualitative inquiry. Survey, open-ended questions. Start 05-12 to 01/11/2012 | Other: The participants were recruited through an authorizationcourse in national subjects | Immigrants nurses from 18 different countries outside the EU. Countries in Asia and Eastern Europe were predominant. | 144 84% women and 16% men, age mean 32. Home country: 18 different countries outside the EU  East Asia/Philippines, EasternEuropa/Serbia and others | Conscientious assisting and proud as nurse Impressed, but challenged as strangers | Resilience as cultural competence which includes: cultural awareness, cultural knowledge, cultural skill, cultural encounter and cultural sensitivity. | | | | | | | | | | |
| Walters, 2008, Australia. Title: The experiences, challenges and rewards of nurses from South Asia in the process of entering the Australian nursing system. | To explore, identify and document, in the words and expressions of participants, the lived experience of immigration, living and working as a nurse in a foreign country. | Narrative analysis. Individual interview. Data collection period not clear | Unclear | Most participants were married and at the time of interview, their families were either with them in Australia or in the process of immigrating to Australia. Their combined nursing experiences included medical, surgical, emergency, neonatal intensive care, orthopaedics, obstetrics, midwifery and psychiatry. | 16 14 females and 2 males, range 26-41. Home country: South Asia | Trust and fear, English language requirements, Immigration, Belonging, integration and family, Living and working in the WesT | ?? From Discussion: Feelings of belonging and familytogetherness filtered through most narratives. Several participants felt they were still in an adjustment phase and many were trying to balance the Eastern/Western lifestyle differences. Participants have an awareness of the extent to which Australian culture willinfluencetheirchildrenbuttheoverridingsensewas one of optimism and opportunity for themselves and their families. | interviews forqualified nurses from other countries should be conducted faceâ€‘toâ€‘face in the source country or via phone or video conferencing. Information regarding Australian lifestyles, culture and working conditions should be conveyed in a timely manner or at interview to enable selected candidates to make informed choices and decisions. Information could include visual aides such as photographs and videos. Selected candidates should be informed as soon as practicable following interview and given realistic time frames for visa processing and position commencement, in order for candidates to appropriately manage their departure activities. | | | | | | | | | |
| Vafeas, 2018, Australia. Title: A heuristic study of UK nurses migration to WA: Living the dream downunder. | To understand the experience of migration for RNs moving from the UK to WA | Heuristic inquiry. Focus group, individual semi-structured interviews, and a journal. Start 2008 to 2011 | Other: notice boards of hospitals and then by snowball sampling | Registered with the Nurses Board of Western Australia had migrated to Western Australia between 2003 and 2008; had previously worked as RNs in the UK, and were employed in the WA healthcare system for at least six months at the time of interview. | 18 females and 3 males, range 26-51. Home country: UK | developing resilience finding a new professional identity having the ability to adapt to a new life | Coping strategies. These qualities incorporated flexibility being able to establish a sense of new personal and professional self-identity; and being able to adjust to change. | Learning from the experiences of past migrants can assist future nurses to â€œlive the dreamâ€ that Australia offers, both professionally and personally. | | | | | | | | | |
| Stubbs, 2017, UK. Title: Recruitment of nurses from India and their experiences of an Overseas Nurses Program. | To explore the transition experiences of nurses recruited from India to London to work in critical care settings in 2011. | A descriptive qualitative approach. Individual interview. Start 11-12 to 01/02/2013 | Clinic | Nurses recruited from India, from three separate hospitals that have critical care units | 11 females and 5 males, age range 25-33. Home country: India | Autonomy and responsibility,  Language,  Culture (food and climate),  Loneliness and work challenges | Autonomous decision making | To provide evidence for recommendations to changes in policy and practice that will be relevant and transferable to any area of a hospital that has Indian nurses undertaking the ONP  Overseas nurses would benefit from being mentored by another nurse from a similar culture, with anon-English background. It may be feasible for overseas nurses to receive training in cultural competencies to improve disparities. |  |  |  |  |  |  |  |  |  |
| Winkelmann-Gleed, 2005, UK. Title: International nursing. Strangers in a British World? Integration of international nurses. | To examines the experiences of recently internationally qualified migrant nurses to Britain and explores their stories with the aim of understanding aspects of their work-related identities | Mixed Method, Survey and interviews. Start 2002 to 2003 | Other: Through self-selection and contacts | Internationally qualified, foreign-born nurses in London. | I:20 females and 2 males S:115 females and 25 men, mean 34,19 years. Home country. Asian, White, Black or Black British | From interview: The perception of migrants by British-trained nurses and patients: hardship Migrant nurses" integration; Carer progression | Experienced fair and respectful interaction | Efforts to meet demands created by nursing vacancies drew a substantial number of migrant nurses to Britain and their Integration is key for the success of care delivery and team relationships. Male as well as migrant nurses form minorities and their perception as strangers' present a challenge to management. Male migrant nurses identify foremost with being migrants, not men. Gender and cultural identities strongly influence work-related ones, however, professional identity and a commitment to career progression is an overriding motivational factor. |  |  |  |  |  |  |  |  |  |
| Fong et.al 2005, Singapore. Title: The expectations and experiences of Myanmar nursing aides working in an inpatient hospice in Singapore. | explore the experience of Myanmar nurses working as nursing aides in the palliativ care setting | Exploratory research. Focus groups. Data collection period not clear | Other: convenience sampling | Myanmar nurses, working as nursing aids in an inpatient hospice in Singapore | 18, sex and age reported. Home country: Myanmar | nursing aids attitude toward life threatening illness stressors coping strategies | Coping strategies | |  |  |  |  |  |  |  |  |  |
| Wheeler, 2014, USA. Title: The experience of discrimination by US and Internationally educated nurses in hospital practice in the USA: a qualitative study. | To document experiences of nurses educated abroad and in the USA in 2 urban hospitals in the southeastern USA. | A qualitative, explorative approach using structuration theory. Individual interview. Start 2011, end unclear | Clinic | Registered nurses from 2 urban hospitals in the southeastern USA. 41 (of 82) were IENs. IENs were raised, received their initial nursing training (leading to licensure) and had at least 1 year of practice in their home countries. US RNs were nurses raised and educated (leading to nursing licensure) in the USA. To be eligible, both IENs and US RNs worked for at least 1 year at the bedside in USA hospitals. | 1. interview :42 IEN and 40 USA educated nurses, all female 2.interview :22 IEN and 18 USRN, all female, US RNs: 39, IENs: 45. Home country: Caribbean, African American, European Union,  Sub-Saharan Africa, Southwest Asia, East Asia,  Pacific, Oceania. | Discrimination by patients, Discrimination by supervisory hospital personnel, Discrimination by fellow nurses, Coping. | Coping: Rely on personal values to help them ignore it,  Excuse the behaviour,  Confront it,  Change units or shifts or  Leave their positions and/or they work harder to prove themselves to everyone around them | More research is needed about discrimination against nurses in the workplace because discrimination may have serious psychological effects that impact nurse retention and the quality of patient care. | | | | | | | | | |
| Kishi, 2014, Australia. Title: A Model of Adaptation of Overseas Nurses: Exploring the Experiences of Japanese Nurses Working in Australia. | To investigate the experiences of Japanese nurses and their adaptation to their work environment in Australia. | Qualitative study. Individual interview. Start 04-2008 to 01/06/2008 | Other: "Purposive and snowball sampling" | Japanese nurse registered nursing experience in Japan study experience in Australia previous or current employment in the NSW health sector  ability to speak English,  willingness to participate in the study | Female: 13, Male: 1, range 30-59. Home country: Japan | Seeking, acclimatizing and settling | Adaptation: their fulfilment and develop their capacity, which is essential for reaching self-actualization or fulfilment | OQNs need better support as they progress to a stage of settling as RNs in a new country. For example, it was clear that identification of appropriate peer mentors was helpful to the participants. | | | | | | | | | |
| Alexis, 2012, UK. Tilte: Exploring the perceptions and work experiences of internationally recruited neonatal nurses: a qualitative study. | The aim of the study was to explore the experiences of internationally recruited neonatal nurses in the NHS in the UK. | Phenomenological approach. Individual interview. Start 08-07 to 01/10/2007 | Other: unclear | IRNNs working in a neonatal unit a minimum of one year but no more than 10 years of experience in a neonatal unit in London. | 13 female, range 24-55. Home country: Jamaica or the Philippines | The support mechanisms,  Unfamiliarity with family centered care,  Feelings of being treated like a child Coping strategies | As coping strategies :they needed to prove that they could cope despite being treated differently. Individuals who feel valued and empowered  Larzarus and Folkman : coping as â€˜constantly changing cognitive and behavioural efforts to manage specific external and internal demands that are appraised as taxing or exceeding the resources of the personâ€™ (p. 141). | Policy makers are key drivers to ensuring that policies are instituted for the benefit of IRNNs and for the sustainability of the workforce. |  |  |  |  |  |  |  |  |  |
| Choi, 2019, New Zealand. Title: Power distance and migrant nurses: The liminality of acculturation. | To explore the transitional discomforts experienced by IQNs, and to identify possible organizational and collegial steps that may ameliorate these challenges | Interpretive phenomenological approach. Individual interview. Start 06-17, end Unclear | Other: Flyers,email or phone | IQNs who obtained their initial nursing registration overseas and then completed their nursing transition through a CAP course in New Zealand; had not worked in other countries as an IQN; had been working as a registered nurse in New Zealand for less than five years; and for whom English was their second language. T | 4 female and 4 male, range 28-34. Home country: 5 Indian; 3 Filipina | Un/learning and the â€˜hidden curriculumâ€™; Destabilisation of expertise; Preceptors and leaders as navigators; Finding oneâ€™s voice | Enthusiastic about the relative egalitarianism they experienced,   Enjoying a newfound level of autonomy, and equity with medical colleagues | Preceptors, managers, educators and migrant nurses themselves  to understand the acculturation process, including the liminal process that is undergone when navigating shifts in power distance, in order to expedite a collaborative and critically reflective approach  to optimising transitions. |  |  |  |  |  |  |  |  |  |
| Jose, 2009, USA. Title: A phenomenological study of the lived experiences of foreign educated nurses working in the United States of America. | Overall aim:To explore and describe the lived experiences of foreign educated nurses (FENs) working in the United States of America  Specific aim to our study since it is a dissertation : Describe strategies the FENs reveal they used to adapt and cope with challenges. | Phenomenological Psychological Method. Individual interview. Data collection period not clear | Other: information were disseminated through e-mail blasts and networking and oral presentations at the meetings of the Filipino nurses association underway snow ball sampling | A purposive sample of 20 full-time employed FENs nurses who migrated to US within the last five years from the countries of The Philippines, India and Nigeria | Male 3 and Female 17, range 23-44. Home country: eight nurses from Philippines, seven nurses from India, and five nurses from Nigeria | Dreams of a better life Difficulties of the journey A shocking reality Rising above the challenges Feeling and doing better Ready to help others | Rinsing above the challenges - building on individual strengths, willing to learn new ways, creating networks, finding support | To contribute to the body of knowledge that supports acculturation of FENs and  add emphasis to the need for US employers to provide cultural and practice-focused education to FENs after they arrive here. In addition, suggestions FENs made about how to help those who plan to come to the US in the future will influence recruitment programs. |  |  |  |  |  |  |  |  |  |
| Eriksson, 2018, Sweden. Title: Internationally educated nurses' and medical graduates' experiences of getting a license and practicing in Sweden - a qualitative interview study. | To describe IENs and IMGs experiences of getting a license to practice and work in the Swedish health and social care system. To evaluate their ability to use their intercultural competence at work, and whether intercultural competence could be an asset in an increasingly multicultural society. | Descriptive in design. Individual interview. Start 10-15 to 01/01/2017 | Other: Human resources departments assisted with contact information for possible participants. Participants received an email and those interested in participating were encouraged to reply by email or phone. Snowball sampling was used to achieve an adequate sample s | Educated inside or outside EU/EEA  A purposive sample | Female: 13 Male: 9, range 35-39. Home country: Bosnia, Bulgaria, Germany, Great Britain, New Zealand, Poland, Serbia, Spain, Sudan, Italy, Syria, Romania, Greece, Uzbekistan | Getting a license -a different story, The work is familiar, yet a lot is new, Trying to master a new language. | Definition of intercultural competence: oneâ€™s ability to communicate effectively and appropriately in intercultural situations based on oneâ€™s intercultural knowledge, skills, and attitudesâ€™ | Organizations and managers employing IENs and IMGs should provide longer workplace introduction to facilitate the acculturation process. More time-efficient language courses specifically adapted to IENs and IMGs could make the transition easier and shorten the time to obtain a license to practice for both profession. |  |  |  |  |  |  |  |  |  |
| Iheduru-Anderson, 2018, USA. Title: Experiences of Nigerian Internationally Educated Nurses Transitioning to United States Health Care Settings. | To characterize the facilitators and barriers to transition of Nigerian IENs (NIENs) to the United States health care setting | Descriptive phenomenology approach. Individual interview. Start 12-13 to 01/02/2014 | Other: Word of mouth, recruitment flyers, e-mails, and telephone calls | To have been educated in Nigeria  prelicensure, be fluent in spoken English, have passed the NCLEX-RN (National Council Licensure Examination) in the United States, and be currently employed as a nurse in  the United States for at least 12 months. | 6 female, mean 39 years. Home country: Nigeria | Fear/anger and disappointment (FAD), Road/journey to success/overcoming challenges (RJO),  Moving forwardâ€ (MF) | Road/journey to success/overcoming challenges : The subtheme of resilience and not giving up includes gaining assertiveness as well as acquiring culturally acceptable behaviors expected in the United States workplace.Empowerment that comes with â€œlearning the systemâ€ and becoming better acquainted with how to live and work in  the United States. | Future research should seek to develop a model for optimal adaptation that focuses on improving both personal and organizational facilitators and decreasing barrier. | | | | | | | | | |
| Eriksson, 2018, Sweden. Title: Internationally educated nurses' descriptions of their access to structural empowerment while working in another country's health care context. | To examine internationally educated nursesâ€™ experiences of empowerment structures using Kanterâ€™s theory of structural empowerment as a framework | A descriptive design. Individual interview. Start 10-15 to 06-2016 | Other: Mail and snowball sampling | Purposive sample all nurses educated abroad | 1 male ,10 female, age 25-59 (35). Home country: Algeria, Bosnia, Bulgaria, Germany, Great Britain, New Zealand, Poland, Serbia, Spain, Sudan | Access to information Access to support  Access to resources Access to opportunities Access to formal power  Access to informal power | empowering structures described in Kanterâ€™s theoryâ€”such as support, information, and informal powerâ€”may help IENs adjust to the new work environment. | Managers need to support IENs when having a team leadership role, facilitate encounters between IENs and ordinary staff, and establishing mentership for IENs. | | | | | | | | | |
| Allen, 2018, USA. Tilte: Experiences of internationally educated nurses holding management positions in the United States: Descriptive phenomenological study. | To explore the experiences of internationally educated nurses in management positions in the US health care organizations to understand the obstacles and support these individualsâ€™ experience when pursuing and working in managerial roles. | Descriptive phenomenological design. Individual interview. Data collection period not clear | Other: Participants were recruited through flyer distribution, email sent by leaders of nursing organisations, and oral invitations | Individuals who earned their initial registered nurse licence outside of the United States, had worked as managers for at least 1 year | 5 females. 2 males, range 30-65. Home country: Philippines 2, India 3, China 1, Jamaica 1 | 1. Supervisors in IENsâ€™ acceptance; 2. Job challenges/responsibilities; 3. Cultural differences; 4. Language and communication; 5. Work relationships and support; and 6. Educational opportunities | The participants were able to overcome the challenges through the support of organizational leaders and the desire to further their education and skills. Volunteering to serve on committees provided opportunities to expand their knowledge and skills, as well as to network with individuals in other areas of the organization. Some participants perceived that actively participating in committees contributed to their success. | The findings indicate that internationally educated nurses may benefit from joining organizational committees, establishing relationships with supervisors, and pursuing education. | | | | | | | | | |
| Al-Hamdan, 2015, UK. Title: Experiencing transformation: the case of Jordanian nurse immigrating to the UK. | This study explored how Jordanian nurses experienced the transition from home to host country to illuminate the elements of transformation. | Unclear. Individual interview. Start unclear, interview conducted over a 6-month period | Other: Through the Nursing and Midwifery Council (NMC) and via snowball sampling. | Jordanian migrant nurses. | 20 males, 5 females, unclear, 11 <30 years, 13: 31-40 years, 1>40 years. Home country: Jordan | Professional transformation, personal transformation and sociocultural experiences | No clear definition, focusing on transformation. â€˜Professional transformationâ€™ as the means of fulfilling oneâ€™s nursing potential by directing oneself towards adding more value to nursing. A personal transformation is used here to identify a shift in thought or action to fulfil a personâ€™s potential by directing the self towards making a difference in their own work and life | To provide high-quality nursing care, it is critical to under-stand the transformation experience to expand our sense of self as a person and as a nurse, gaining a degree of control over how we can practise when we move anywhere in the world. | | | | | | | | | |
| Salami, 2018, Canada. Title: Downward occupational mobility of baccalaureate-prepared, internationally educated nurses to licensed practical nurses. | The experience of baccalaureate-prepared, internationally educated nurses who work as licensed practical nurses in Canada | Exploratory transnational feminist qualitative research design. Individual interview. Start 03-16 to 11-2016 | Other: Through a database compiled during an earlier research project conducted by the third co-author; and snowball sampling | Have earned a baccalaureate degree in nursing prior to migrating to Canada and must be currently working as an LPN in Canada or have worked as an LPN within the past 2 years. | 12 females, 2 males, range 27-52. Home country: Philippines (N= 9), India (N=3), Nigeria (N=1), Mauritius (N=1) | Hope for a better personal and professional life;  Barriers to workforce integration as registered nurses and discover an easier path in the licensed practical nurse registration process;  Deskilling and ambivalent skill recognition;  Dissatisfied as a licensed practical nurse in Canada. | Hope: Participants were deeply motivated to provide a better future for their kids and to enable their children to have better educational opportunities. Thus, familial responsibility as mothers served as an important motivation that propelled the migration of this group of nurses. Participants also experienced unfavorable working conditions and poor pay in the country of origin, and so hoped to improve their lives with migration | Implications for policymakers and educators to include the need to address the barriers to becoming registered nurses, including application processing times and lack of adequate access to educational programmes. | | | | | | | | | |
| Connor, 2016, USA. Title: Cultural Influence on Coping Strategies of Filipino Immigrant Nurses. | Discusses the strategies that Filipino IENs  use to cope effectively with their work-related and nonwork-related stress explored stress and coping in a sample of  A part of a larger study :Filipino IENs within  the context of the immigration and adaptation process | A cross-sectional qualitative descriptive  design. Individual interview. Data collection period not clear | Other: Network as professional colleagues, leaders from Filipino nursing  associations and social and religious organizations, and personal acquaintances who worked with IENs or were well connected with the target population. Then telephone | Filipino women who (a) were at least 21 years old, (b) received their basic nursing education in the Philippines, (c) worked as a registered  nurse (RN) in the Philippines prior to migrating to the United States, and (d) worked in a U.S. health care facility at the time of the interview. | 20 female, range 28-48. Home country: The Philippines | (a) familial coping , (b) intracultural coping , (c) fate and faith-based coping, (d) forbearance (patience and self-control) and contentment , (e) affirming the nursing profession and proving themselves , and (f) escape and avoidance | Coping is defined as the individualâ€™s cognitive and behavioral responses to manage the internal and external demands of stress (Folkman, 2013; Lazarus & Folkman, 1987) | Occupational health nurses and administrators can use these findings to develop culturally appropriate health promotion programs and interventions to retain quality nurses and  promote healthier workplaces. |  |  |  |  |  |  |  |  |  |
| Healee, 2016, New Zealand. Title:Working with difference: Thematic concepts of Japanese nurses working in New Zealand. | The purpose of this study was to compare the differences experienced by Japanese nurses working in NewZealand from an organizational and personal perspective | A qualitative descriptive design. Individual interview. Data collection period not clear | Other: Intermediary,given information sheets, and asked to contact one of the researchers to volunteer. | Japanese nurses, employed within the public health system hospital setting or a residential care facility, had been born in Japan, and had immigrated to NewZealand over five years ago. | 9 female, Age range 30-40, Home country: Japan | Finding a voice Two subthemes: accommodating difference, and learning to speak up | Finding a voice was a method of negotiating how they practised nursing in New Zealand compared to Japan.   To learn to accommodate difference while learning to speak up | Managing this transition well will enable nurses to appreciate the richness diversity brings to the profession. | | | | | | | | |  |
| Rodriguez, 2014, Chile. Title: Cultural experiences of immigrant nurses at two hospitals in Chile. | To explore the cultural experiences of nurses who immigrated to Chile. | Ethnography. Observation-Participation-Reflection Model. Observation and interview. Start 03-10 to 08-2010 | Other: the fact that the primary author of the study was a nurse at one of the institutions studied and an immigrant in Chile, contact established by phone or in person | Being an immigrant nurse, graduated in the country of origin, at least one year of professional practice in Chile, having  a job contract in one of the two cultural contexts and  being willing to cooperate with this study. | 15 female, Age range 24-44. Home country: Brazil (1), Colombia (7), Ecuador (3) and Uruguay (4) | In search of better horizons â€“ the decision to immigrate to Chile; Gaining trust and establishing a support network â€“ employability and professional performance; Seeking people Ìs acceptance â€“ professional adaptation in a new cultural scenario | Professional development aspirations  Better living conditions and quality of life  Help from family members,friends,boyfriends or husbands The main facilities involved the existence of  job opportunities, some similarities in the professional  education and support from patients. | The professionals need to be offered support  during the first year of their study, through educative  programs and interventions aimed at achieving progress  in their cultural awareness and at improving their level of competence |  |  |  |  |  |  |  |  |  |
| Lin, 2014, USA. Title: Filipina nurses' transition into the US hospital system. | Exploring how Filipina nurses transition into their role as nurses and adapt to nursing practice in the US. | Qualitative approach. Individual interview. Start 11-06 to 01-2009 | Other: Word-by-mounth | A sampling of emigrant Filipina RNs employed in a hospital setting | 31 females, age not reported. Home country: Philipines | Conceptualizing US nursing, reacting and interacting | Focus on transition from one culture to another, to ease the transition process: Seek help from others, learn to face/deal with it, Seek spiritual support, Seek support from others, Mingle with other Filipinos, Maintain open-minded attitudes, Learn from Filipino mentors, Embrace the US culture, Learn to speak up/be assertive | customized orientation programs and soliciting new emigrant hire feedback, should acceleratesâ€™ adjustment and ease their role transitions | | | | | | | | | |
| Wolcott, 2013, USA. Title:Integration of internationally educated nurses into the U.S. Workforce. | To explore the experiences of internationally educated nurses and the nurse managers and educators working with them, to understand the issues, and to highlight potential solutions for addressing integration challenges. | Grounded theory. Individual interview. Data collection period not clear | Other: mail, flyers and snowball sampling technique | Nurses currently practicing in Northern California: (a) those whose nursing education was obtained outside the United States (IENs), (b) nurse educators who assess and educate new IENs, and (c) nurse managers who hire and evaluate IENs. | 13: 5 IEN 4 Educators 4 Managers, sex and age not reported. Home country: Denmark, Germany, India, Philippines, and Portugal. | Communication difficulties; financial challenges; the need for outside social support; and educational orientations focused on culture, nurse role, and communication techniques. | Royâ€™s adaptation model as a theoretical framework, the four modes of adaptation were used to explore experiences of the integration process for IENs, nurse educators, and man agers. Roy defined adaptive as the humanâ€™s capacity to adjust effectively to changes in the environment and affect the environment. | Prepare current staff for arrival of IENs; - provide cultural sensitivity training for managers, educators and all nursing, medical, and ancillary staff, including IENs; - develop orientation programs that include an introduction to U.S. healthcare culture, the nursing process, assertiveness training, advocacy, and critical thinking; - initiate preceptorships and mentorships formally or informally for IENs, both on the job and in a social arena; - provide initial fiscal support in terms of housing and spending money; - encourage language classes for those new to English that focus on medical/nursing vocabulary beyond simple conversational English; consider accent reduction; - implement team-building activities and identification of shared values to ensure long-term efficacy, integration, and retention |  |  |  |  |  |  |  |  |  |
| Ronquillo, 2012, Canada. Title: Leaving the Philippines: oral histories of nurses' transition to Canadian nursing practice. | This study examines the transition experiences of Filipino nurses who immigrated to Canada between 1970 and 2000 | Oral history research methodology. Individual interview. Start 12-09 to 05-2010 | Participants were recruited through purposive and snowball sampling. | Filipino nurse immigrants residing in the provinces of Alberta and British Columbia | 9 females Participants ranged in age from early thirties to late fifties. Home country: Philippines | Family First, Nursing Later Nursing in Canada: Different Expectations Being Foreign: Proving Oneself and Perceptions of Discrimination | Immigration journey, transition experiences Familial responsibility and sacrifice, shifting expectations of what it means to be a nurse in Canada, and perceptions of â€œfeeling foreignâ€ and the need to prove oneself were common themes. |  |  |  |  |  |  |  |  |  |  |
| Salma, 2012, Canada. Title: Career advancement and educational opportunities: experiences and perceptions of internationally educated nurses. | To encourage ethical IEN employment by understanding their needs, and (b)  To maintain successful IEN recruitment by understanding factors which increase retention | An interpretive descriptive methodology. Individual interview. Start uncelar, interview conducted over 4 months | Other: recruitment posters were distributed within two hospital  Nursing colleagues introduced the research three participants were recruited through other participants Snowballing | Registered IEN nurses with at least two years of experience in Canadian healthcare organizations both long-term care and hospital | 1 male and 10 females, two participants were between 20 and 30 years of age.  Three participants were between 30 and 40 years of age. eight participants were above 45years of age. Home country: Guyana, China, Iran, Philippines, India, Britain, and New Zealand. | Motherhood as a priority, communication challenges,  learning a new culture,  the process of skill recognition,  perceptions of opportunity,  personal responsibility for success, personal definition of advancement, and need for mentorship | Being a mother for my children seeking financial stability,  Pride Need for financial support Skill recognition Personal responsibility, resilience, and personal motivation for success.  â€œup to youâ€, â€œsurpassâ€, â€œadaptâ€, â€œyou canâ€, and â€œyou have toâ€when talking about success in the workplace. | This study calls for is a commitment in both policy and practice to creating equitable opportunities in career and education advancement, not only for IENs, but for all visible minority groups within the Canadian context. |  |  |  |  |  |  |  |  |  |
| Bland, 2011, New Zealand. Title: From India to New Zealand--a challenging but rewarding passage. | This study, sought to explore the experiences of Indian RNs who, after completing a New Zealand RNBN programme in India, worked in New Zealand as RNs | Qualitative inquiry, informed by participatory action research. Focus group. Start 2005 to 2008 | Other: The research was introduced to potential participants by a person not involved in the study. Twenty-two nurses who had com-pleted or were close to completing the UCOL RNBN programme in India, and who planned to migrate to New Zealand, were invited to participate | Indian RNs who, after completing a New Zealand RNBN programme in India, worked in New Zealand as RNs. | 7 women, 3 men, Age range 23-35. Home country: India | 1.To find something better for themselves - a desire to improve their nursing knowledge and skills,  2. Better employment opportunities. | Unclear, working in a foreign country was expected to lead to better working conditions, greater access to health-related technology and higher remuneration. This particular group of nurses were positive their dream of a better future was on the way to being realised: it was a dream of one or two years, it was a dream of a whole future, so it is still progressing, and so far it is good. | To ensure the safe passage of all IRNs who  come to NZ, supporting their integration into the workforce and maximising their potential contribution to health care. |  |  |  |  |  |  |  |  |  |
| Magnusdottir, 2005, Iceland. Title: Overcoming strangeness and communication barriers: a phenomenological study of becoming a foreign nurse. | To generate an understanding of the foreign nurses experience with the purpose of contributing to a constructive international nursing/health care climate in Iceland. | Phenomenology ;a hermeneutic methodology influenced by constructivism. Individual interview. Data collection period not clear | Other: Sampling was purposeful, where the task was to endorse maximum phenomenal and demographic variation. | Foreign nurses working at hospitals in Iceland | 11, sex not described, age range 20-35. Home country: 7 western and 4 non-western | First theme: tackling the initial, multiple challenges Second theme: becoming an outsider and the need to be let in Third theme: struggling with the language barrier Fourth theme: adjusting to a different work culture Fifth theme: overcoming challenges to win through | Nearly all felt they had grown through the experience. They spoke about being stronger, more independent and having better self-knowledge. Some felt they were more open-minded, more expressive and softer persons. | The findings and their international context suggest the importance of language for personal and professional well-being and how language and culture are inseparable entities. | | | | | | | | | |
| Liou, 2011, USA. Title: Experiences of a Taiwanese nurse in the United States. | To explore and interpret the lived experience of a Taiwanese nurse working in a U.S. hospital | Combining hermeneutic phenomenology with the case study approach. Individual interview. Data collection period not clear | Other: For recruitment, the author canvassed her colleagues for the name of prospective participants. From the referrals, the author was able to identify an individual who met the selection criteria and who consented to participate in the study. | To (a) be born and have gotten nursing education in an Asian country, (b) be employed as a registered nurse in a U.S. healthcare facility, and (c) be willing to participate in this study | 1 female. 35 years. Home country: Taiwan | (a) frustration in language and communication; (b) cultural differences in patient care; (c) support from work environment; and (d) advantages of working in the U.S. nursing system. | support from many of her nurse colleagues and some patients. This support helped ease her hurt feelings and gave her energy to move forward in her job. Human rights, patient -nurse ratio | Relevant to nurses and administrators who work with international nurses who wish to understand international nursesâ€™ frustration, needs, and work values |  |  |  |  |  |  |  |  |  |
| Jose, 2011, USA. Title: Lived experiences of internationally educated nurses in hospitals in the United States of America. | To elicit and describe the lived experiences of internationally educated nurses (IENs) who work in a multi-hospital medical centre in the urban USA. | Phenomenology. Individual interview. Data collection period not clear | Other: Recruitment materials were sent to a Registered Nurse list serve and posted in the nursesâ€™ rest areas on hospital units. Once data collection was underway, snowball sampling, where current participants referred the prospective participants, was also used. | The final study sample consisted of20 IENs: 8 from The Philippines, 7 from India and 5 fromNigeria as the source countries. | 3 Male and 17 female, Participants ranged in age from 23 to 44 years with an average of 33.2 years. Home country: The Philippines (8), India (7) Nigeria | (1) dreams of a better life,  (2) a difficult journey,  (3) a shocking reality,  (4) rising above the challenges,  (5) feeling and doing better (6) ready to help others | Individual strengths were emphasized within the context of rising above the challenges The positive work ethic of IENs, their persistence, willingness to learn and adapt, and psychosocial and logistical support  Social support offered stress relief and built confidence that helped them to rise above the challenges, feel better, and help others. | The recruiters, employers, staff-development educators, peers and the IENs themselves will find the report of this study useful as they work together to develop and improve strategies and programs that target IENs need. |  |  |  |  |  |  |  |  |  |
| Xu, 2008, USA. Title: Adaptation and transformation through (un)learning: lived experiences of immigrant Chinese nurses in US healthcare environment. | This study examines the lived experiencesof a group of Chinese nurses working inthe US healthcare environment. | Phenomenology. Individual interview. Data collection period not clear | Other: By word of mouth and referral. | Working as registered nurse clinicians and identify themselves as ethnic Chinese. 5 worked as staff nurses, 3 as clinical leaders(ie, advanced clinical nurses who served as resource nurses on the unit because of recognized clinical expertise), and 1 as a supervisor. In terms of their jobs before their arrival in the United States, 4 worked as nurses, 2 as teachers, 1 as a computer software engineer,1 as a secretary, and 1 as a physician. | 9 female mean 40,4 (32-51). Home country: China | (a)Communication as the most daunting challenge, especially during initial transition of their first job; (b) different and even conflicting professional values and roles/expectations of the nurse between the United States and China; (c) marginalization, inequality, and discrimination;(d) transformation through clinging to hope, (un)learning, and resilience; and (e) cultural dissonance. | No clear definition found, but by clinging to hope and adapting through (un)learning and resilience, the Chinese nurses transformed into competent professional nurses and clinical experts, enjoyed their work, and pursued their career dreams trough self-confidence, strength, assertiveness, persistence, and determination; valuing education and life-long learning; taking initiative; never giving up. | this study indicates that they do possess unique strengths that are appealing to and valued by Western countries | | | | | | | | |  |
| Sochan, 2007, Canada. Title: Acculturation and socialization: voices of internationally educated nurses in Ontario. | This study explored the experiences of IENs in their journey to become licensed RNs in Ontario. A number of questions guided this exploration. What are their personal stories? Are their experiences similar? Is there atypical journey to become an RN in Ontario? | A Biographical Narrative (qualitative) research methodology. Individual interview. Start 06-05 to 09-2005 | Other: A convenience sample of 12 students from a community college volunteered for this study. | Volunteer IEN students enrolled in bridging programmes within a large community college in the Greater Toronto Area were recruited for this study | 1 male and 11 female, age not reported. Home country: the Philippines (5), Mainland China (2), India (2), South Korea (2) and Ukraine (1). | The Told portion of this overall theme, including: (a) wanting the Canadian dream (of becoming an Ontario RN); (b) discovering that their home-country nursing qualification does not meet Ontario entry to practice; and (c) the redefined Canadian dream of returning to nursing school to upgrade their nursing qualifications.  Untold interpretations of these three themes are: (a)hope, (b) disillusionment, and (c) navigating disillusionment. | Hope: wanting the Canadian dream of becoming an RN in Ontario. Useful qualification for Canada, and further be able to practise as nurses in Ontario. Fulfilment of a dream   Navigating disillusionment: To accept their fate, and have reconciled themselves to returning to school to upgrade their nursing qualifications to meet Ontario entry to practice requirements. Viewed this journey with an ironical sense of humour. | IEN recommendations for competency assessment: Standardize and streamline the process. Communicate changes in the process: to the academic institutions of higher learning where nursing is taught, to the public and private agencies recruiting IENs.   IEN recommendations for IEN bridging programmes: Identify and address the learning needs of IENs relevant to the Canadian context of nursing practice. Design/deliver courses that facilitate teaching/learning of multicultural and multilingual students. Consider the financial constraints of IENs. |  |  |  |  |  |  |  |  |  |
|  |  |  |  |  |  |  |  | Supporting methods are used to enhance comprehension and learning, for example visual, demonstration,  interactive and written; Greater emphasis, identification and understanding are  required in relation to the purpose and function of the NMC (NMC, 2002) and to the role of the overseas nurse;  Cultural issues and awareness sessions should be used; Greater emphasis should be given to communication and language sessions within the sphere of practice; Overseas nurses should have the opportunity to check concepts, seek clarification and relate the information to  ensure effective communication and learning take place; Support and increased understanding of the purpose and role of the adaptation programme for interdisciplinary staff to promote effective working relationships,  improve morale and a greater understanding and respect for each otherâ€™s cultures. |  |  |  |  |  |  |  |  |  |
|  |  |  |  |  |  |  |  | Recommending policy changes The need for a fair process of hearing complaints and redressing grievances to uphold equal access and opportunity |  |  |  |  |  |  |  |  |  |
|  |  |  |  |  |  |  |  | Concepts as adjustment, mastering, problem-solving strategies and intercultural effectiveness are discussed. | | | | | | | | |  |
